# Diversity of the *Ry*_*sto*_ gene conferring resistance to potato virus Y in wild relatives of potato

**DOI:** 10.1186/s12870-024-05089-2

**Published:** 2024-05-08

**Authors:** Paulina Paluchowska, Simeon Lim Rossmann, Erik Lysøe, Marta Janiszewska, Krystyna Michalak, Rasoul Heydarnajad Giglou, Mousa Torabi Giglou, May Bente Brurberg, Jadwiga Śliwka, Zhimin Yin

**Affiliations:** 1 Plant Breeding and Acclimatization Institute - National Research Institute (IHAR-PIB) in Radzików, Młochów Division, Platanowa St. 19, Młochów, 05-831 Poland; 2 Department of Plant Sciences, Norwegian University of Life Sciences (NMBU), Ås, Norway; 3 Division of Biotechnology and Plant Health, Norwegian Institute of Bioeconomy Research (NIBIO), Ås, Norway; 4 Department of Horticultural Sciences, Faculty of Agriculture and Natural Resources, University of Mohaghegh Ardabili, Ardabil, 56199–11367 Iran

**Keywords:** Amplicon sequencing, AmpSeq, Extreme resistance, *Hyoscyamus niger*, PacBio, *Physalis peruviana*, PVY, *Solanum*

## Abstract

**Background:**

Potato virus Y (PVY) is among the economically most damaging viral pathogen in production of potato (*Solanum tuberosum*) worldwide. The gene *Ry*_*sto*_ derived from the wild potato relative *Solanum stoloniferum* confers extreme resistance to PVY.

**Results:**

The presence and diversity of *Ry*_*sto*_ were investigated in wild relatives of potato (298 genotypes representing 29 accessions of 26 tuber-bearing *Solanum* species) using PacBio amplicon sequencing. A total of 55 unique *Rysto-like* sequences were identified in 72 genotypes representing 12 accessions of 10 *Solanum* species and six resistant controls (potato cultivars Alicja, Bzura, Hinga, Nimfy, White Lady and breeding line PW363). The 55 *Rysto-like* sequences showed 89.87 to 99.98% nucleotide identity to the *Ry*_*sto*_ reference gene, and these encoded in total 45 unique protein sequences. While *Rysto-like26* identified in Alicja, Bzura, White Lady and *Rysto-like16* in PW363 encode a protein identical to the Ry_sto_ reference, the remaining 44 predicted Rysto-like proteins were 65.93 to 99.92% identical to the reference. Higher levels of diversity of the *Rysto-like* sequences were found in the wild relatives of potato than in the resistant control cultivars. The TIR and NB-ARC domains were the most conserved within the Rysto-like proteins, while the LRR and C-JID domains were more variable. Several *Solanum* species, including *S. antipoviczii* and *S. hougasii*, showed resistance to PVY. This study demonstrated *Hyoscyamus niger*, a *Solanaceae* species distantly related to *Solanum*, as a host of PVY.

**Conclusions:**

The new *Rysto-like* variants and the identified PVY resistant potato genotypes are potential resistance sources against PVY in potato breeding. Identification of *H. niger* as a host for PVY is important for cultivation of this plant, studies on the PVY management, its ecology, and migrations. The amplicon sequencing based on PacBio SMRT and the following data analysis pipeline described in our work may be applied to obtain the nucleotide sequences and analyze any full-length genes from any, even polyploid, organisms.

**Supplementary Information:**

The online version contains supplementary material available at 10.1186/s12870-024-05089-2.

## Background

Potato (*Solanum tuberosum* L.) is the third most important food crop of the world after rice and wheat [1]. More than 50 viruses can infect potato, of which *Potato virus Y* (PVY, genus *Potyvirus*, family *Potyviridae*) is among the economically most damaging ones with tuber yield losses up to 80% [2]. Dupuis et al. (2023) estimated that the annual economic loss due to PVY in Europe was EUR 187 million [3]. Recent studies have revealed the emergence of damaging recombinant strains of this virus [4–6].

Wild relatives of potato are sources of resistance genes that confer resistance to many pathogens, including PVY [1, 7, 8]. Extreme resistance conferred by the dominant *Ry* genes protects potato plants against all strains of PVY and suppresses virus multiplication in inoculated cells [9, 10]. Three different *Ry* genes have been identified: *Ry*_*sto*_ from *Solanum stoloniferum* [11], *Ry*_*adg*_ from *S. tuberosum* ssp. *andigena* [12, 13], and *Ry*_*chc*_ from *S. chacoense* [14]. *Ry*_*chc*_, *Ry*_*adg*_ and *Ry*_*sto*_ were mapped to chromosomes IX, XI, and XII of the potato genome, respectively [14–17]. The *Ry* genes have been used in potato breeding programs worldwide and are introgressed into cultivars – like Assia, Barbara, Bzura, Cordia, Hinga, Jumbo, Klepa, Maxi, Meduza, Nimfy, Omulew, Pirola, Ute, Wega, and White Lady. The *Ry*_*sto*_ gene was shown to present in 54 extremely resistant European potato cultivars from Germany, Hungary, Poland, and The Netherlands [15, 18]. So far, only two *Ry* genes have been cloned, *Ry*_*sto*_ from the dihaploid clone of potato cultivar Alicja, which has the PVY-resistant *S. stoloniferum* (breeding clone MPI 55.957/54) in its ancestry [19], and *Ry*_*chc*_ from *S. chacoense* [20, 21]. *Ry*_*sto*_, the first cloned *Ry* gene, encodes a nucleotide-binding leucine-rich repeat (NB-LRR) protein with an N-terminal Toll/interleukin receptor-like (TIR) domain [19]. Ry_sto_ recognizes the coat protein of PVY and other potyviruses, and it confers immunity to plum pox virus and turnip mosaic virus in both *Solanaceae* and *Brassicaceae*, indicating its potential use in various crops against potyviruses [22].

The aim of this study was to analyze diversity and distribution of *Ry*_*sto*_ homologues in 26 tuber-bearing *Solanum* species. We used single molecule real time (SMRT) PacBio sequencing, for obtaining circular consensus sequences (CCS) of high accuracy from multiple long amplicons covering the whole gene. Two *Solanaceae* species *Hyoscyamus niger* L. and *Physalis peruviana* L., which are distantly related to *Solanum*, were included. Both species are used as herbal medicine [23, 24], and *P. peruviana* is grown for its edible berries. Selected species, accessions and genotypes were tested for resistance against PVY. We describe 53 new and diversified variants of the *Ry*_*sto*_ gene in mostly Mexican *Solanum* spp.

## Results

### PVY resistance of *Solanum* species, *Hyoscyamus niger* and *Physalis peruviana*

For three *Solanum* accessions all genotypes were resistant to PVY, i.e., there was no infection in the upper non-inoculated leaves of any tested plants according to enzyme-linked immunosorbent assay (ELISA): *S. antipoviczii* accession 333099 (five genotypes), *S. neoantipoviczii* accession 333117 (six genotypes) and *S. polytrichon* accession 333108 (two genotypes) (Table S1). In the *S. hougasii* accession 333148 resistance to PVY was segregating; four genotypes were resistant, and one genotype was susceptible. *Solanum* accessions 333119 (*S. aemulans*, two genotypes), 333150 (*S. aracc-papa*, two genotypes), 333110 and 333112 (*S. fendleri*, eight genotypes), 333147 and 333159 (*S. papita*, 15 genotypes) and 333157 (*S. verrucosum*, seven genotypes) were all susceptible to PVY (Table S1).

**Fig. 1:**
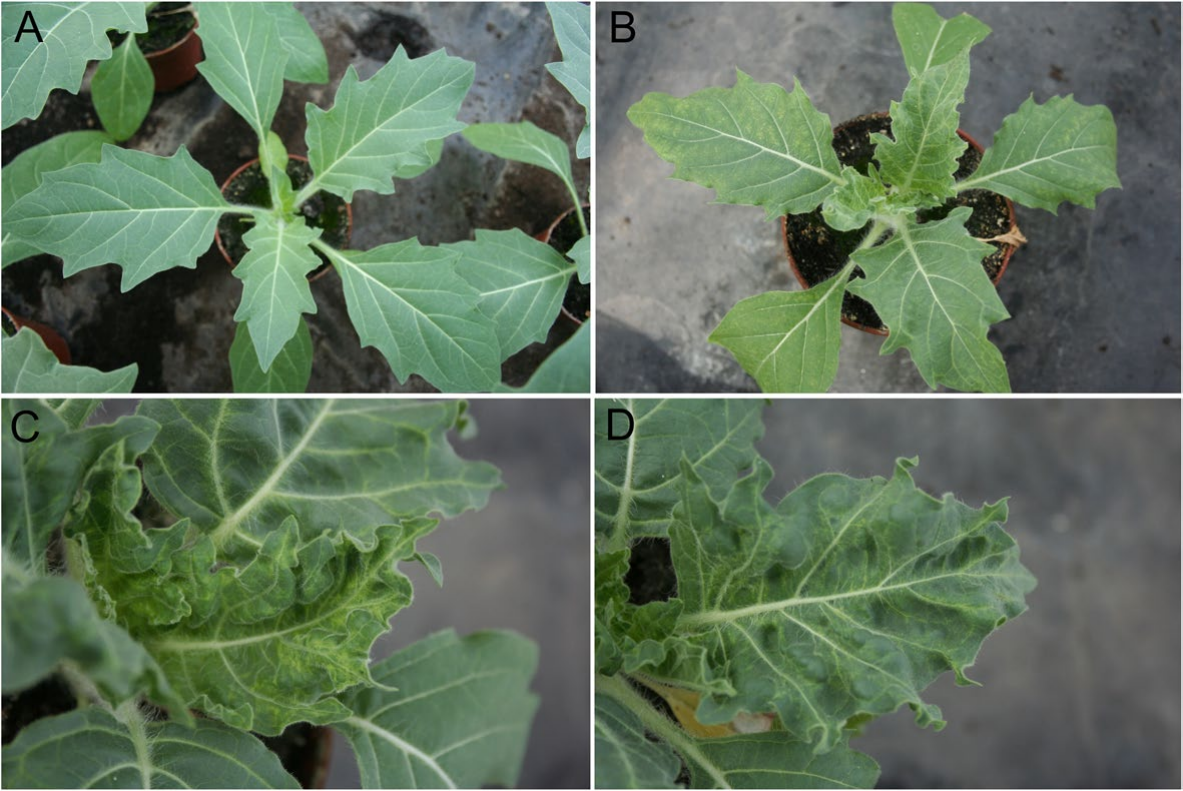
Potato virus Y symptoms in *Hyoscyamus niger* plants following mechanical inoculation with the virus. **A** Healthy plant. **B**, **C** and **D** Symptoms in the upper (non-inoculated) leaves of PVY inoculated plants. In (**B**): Dwarfing, leaf deformation, chlorotic and necrotic spots on leaves. In (**C**): Severe leaf deformation, severe chlorotic spots on young leaves and severe vein-yellowing. In (**D**): Leaf deformation, chlorotic spots on leaves and vein-yellowing. The size of the pots used in (**A**) and (**B**) is the same. Photos were taken 14 days post inoculation

*Hyoscyamus niger* plants were susceptible to all PVY strains and showed severe symptoms, including dwarfing, leaf deformation, chlorotic and necrotic spots on leaves and vein-yellowing (Fig. 1). Virus infection was confirmed in the upper non-inoculated leaves of all tested *H. niger* plants at 14 days post inoculation (dpi) using ELISA (Table S1). *P. peruviana* showed strain-specific resistance to the PVY^O^ strain. The other four strains PVY^NTN^, PVY^N−Wi^, PVY^E^ and PVY^Z^-NTN caused infection in at least half of the tested *P. peruviana* plants, and only mild symptoms were observed in some infected plants.

### Detection of fragmentary and full coding regions of *Ry*_*sto*_

Initially, 298 genotypes representing 29 accessions of 26 *Solanum* species listed in Table S2 were tested for the presence of *Ry*_*sto*_ gene homologues by targeting three fragments of the gene by PCR amplification using primer pairs A, C and J. Fragments of the *Ry*_*sto*_ homologues were detected in 108 of the 298 tested genotypes, including 102 genotypes representing 12 accessions of 10 tuber-bearing *Solanum* species and six resistant potato control genotypes (cultivars Alicja, Bzura, Hinga, Nimfy and White Lady, and the breeding line PW363), but not in the susceptible potato cultivars Irga, Irys, Nicola and Vineta (Figure S1, A and Table S3).

In the next stage, amplification of the entire coding region of the *Ry*_*sto*_ homologues was attempted from the 108 genotypes in which putative fragments of *Ry*_*sto*_ homologues were detected. Three alternative primer pairs T, U, and V were used to increase the chances of amplification of the full coding region of the *Ry*_*sto*_ homologues (Figure S1 D). The full coding region of *Ry*_*sto*_ homologues were detected in 83 plant genotypes, including 77 genotypes representing 12 accessions of 10 tuber-bearing *Solanum* species and the six resistant controls (Figure S1, B and Table S3). Amplification of the full coding sequences of *Ry*_*sto*_ homologues from different *Solanum* species showed primer-specificity. For 25 of the genotypes, the full coding regions were obtained only by primer pair T, which did not include the ATG start codon. The full coding regions including start codon were obtained for 32 genotypes by primer pair V and nine genotypes using primer pair U. For the remaining genotypes, the full coding regions were obtained by two or three primer pairs. Afterwards, 91 barcoded amplicons covering full length of the *Ry*_*sto*_ gene were obtained from 81 plant genotypes (Figure S1, C and Table S3).

### Variants the *Ry*_*sto*_ homologues

**Fig. 2:**
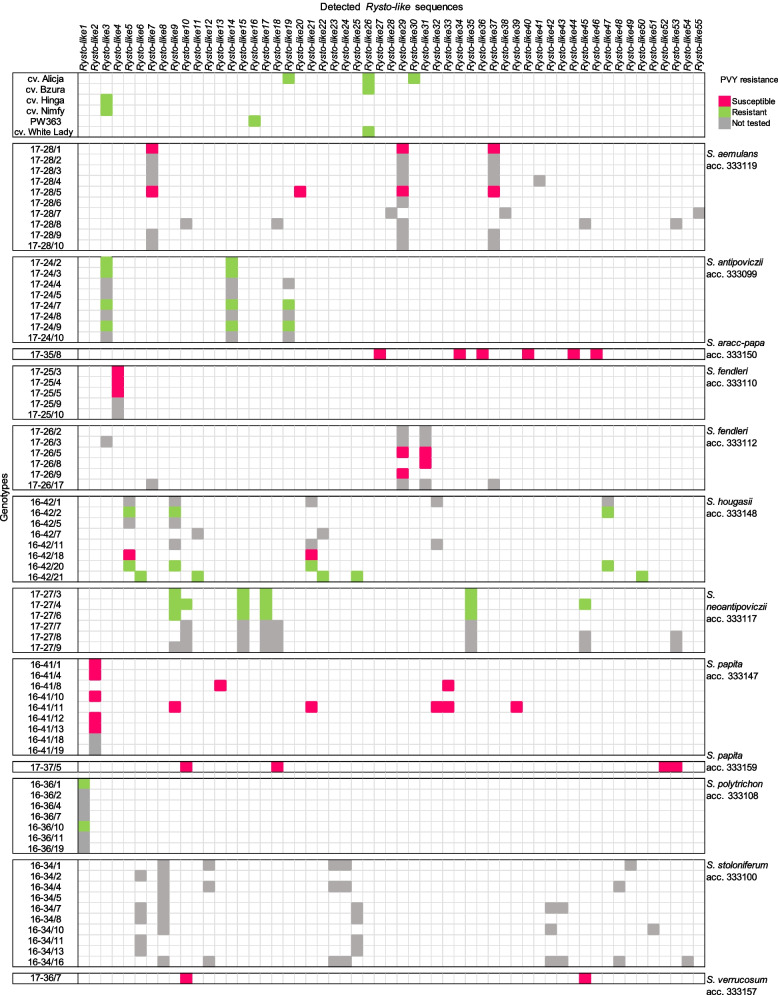
PacBio amplicon sequencing of *Ry*_*sto*_ homologues in *Solanum* species. Fifty-five *Rysto-like* sequence variants (*Rysto-like1* to *Rysto-like55*) were obtained from 72 potato genotypes (17–28/1 to 17–36/7) representing 12 accessions (acc.) 333119 to 333157 of 10 tuber-bearing *Solanum* species (*S. aemulans* to *S. verrucosum*) and six resistant controls (potato cultivars Alicja, Bzura, Hinga, Nimfy, White Lady and breeding line PW363)

In total, 1,761,425 HiFi reads were obtained from PacBio sequencing, and these were from 88 of the 91 barcoded amplicons (Table S4). After removing the 5' and 3' UTRs manually, a total of 55 unique sequences were identified in 78 plant genotypes (Fig. 2 and Tables S3). Annotation using FGENSH revealed five exons and four introns for all 55 sequences, hence the *Ry*_*sto*_ reference gene (MN393235.1; 4762 bp) isoform with the same exon and intron structure was used as a reference. The 55 sequences showed 89.87 to 99.98% nucleotide identity to the reference *Ry*_*sto*_ gene. Therefore, they were named *Rysto-like1* to *Rysto-like55* (Table S5).

The 55 *Rysto-like* variants were detected in 10 *Solanum* species (12 accessions, 72 genotypes) (Fig. 2, Table S3). From one to 12 variants were detected per accession, and between one and eight per potato genotype (Table S3). For the *Solanum* species in which resistance to PVY was confirmed in this study, the number of *Rysto-like* sequences were 10 in *S. hougasii*, eight in *S. neoantipoviczii*, three in *S. antipoviczii* and one in *S. polytrichon*. In *S. stoloniferum*, in which resistance to PVY was demonstrated previously [8], in total 12 *Rysto-like* variants were found.

In four genotypes, 17–27/1 and 17–27/2 (*S. neoantipoviczii*), 17–24/6 (*S. antipoviczii*) and 16–42/22 (*S. hougasii*), resistance to PVY was confirmed by ELISA and fragments of the *Ry*_*sto*_ gene were detected by PCR, but the complete coding region could not be amplified (Table S3).

### *Rysto-like* variants in the resistant potato controls

In each of the resistant potato controls, the number of *Rysto-like* sequences varied from one to three (Fig. 2 and Table S3). For cultivar Alicja, three *Rysto-like* sequences (*Rysto-like19*, *Rysto-like26*, and *Rysto-like30*) were obtained using barcoded primers V, while only one, *Rysto-like26*, was obtained using the barcoded primers U. For the remaining five resistant control genotypes, only the barcoded U primer pair was applied, and a single *Rysto-like* sequence was obtained for each cultivar, i.e., *Rysto-like26* in Bzura and White Lady, *Rysto-like3* in Hinga and Nimfy, and *Rysto-like16* in breeding line PW363.

Based on the obtained *Rysto-like* sequences, the resistant controls used in this study can be divided into three groups. The first group contains potato cultivars Alicja, Bzura and White Lady, where the *Rysto-like26* was detected, showing 99.98% nucleotide identity to the *Ry*_*sto*_ reference gene (MN393235.1) (Table S5). For the *Rysto-like26*, the deletion of one nucleotide (T) compared to the reference was observed in the third intron. The deletion is in a poly-T_18_ tract, position 5243–5260 in the *Ry*_*sto*_ reference, giving poly-T_17_ in *Rysto-like26*. The same deletion of a single T was also noted in *Rysto-like3* and *Rysto-like16* and it was additionally confirmed in cultivars Bzura, Nimfy, Hinga, White Lady and the breeding line PW363 by targeted Sanger sequencing of this region. Two additional *Rysto-like* sequences found in cultivar Alicja were *Rysto-like19* and *Rysto-like30*, with 93.69% and 95.08% nucleotide identity to the *Ry*_*sto*_ reference gene, respectively (Table S5). The *Rysto-like19* was also detected in five of the eight tested genotypes of *S. antipoviczii*. The second group of resistant controls contains the breeding line PW363, where *Rysto-like16* showed 99.96% nucleotide identity to the reference gene (Table S5). This variant differs from the reference sequence by two nucleotides, the deletion in the poly-T in the third intron described above and a nucleotide substitution 5248 T > G within the poly-T tract in the third intron. The third group includes potato cultivars Hinga and Nimfy in which *Rysto-like3* was detected. In addition to the changes in the third intron mentioned above (substitution and deletion), there were two single nucleotide substitutions, 4251A > T (synonymous) in the second exon and 5650C > A (non-synonymous) in the fourth exon. The latter resulted in a single amino acid substitution 746A > E. The *Rysto-like3* was also detected in all tested genotypes of *S. antipoviczii* and in one genotype of *S. fendleri* (acc. 333112). The *Rysto-like16*, *Rysto-like26* and *Rysto-like30* variants detected in the resistant potato controls were not found in any of the tested tuber-bearing *Solanum* species.

### Nucleotide diversity of *Ry*_*sto*_gene homologues

**Fig. 3:**
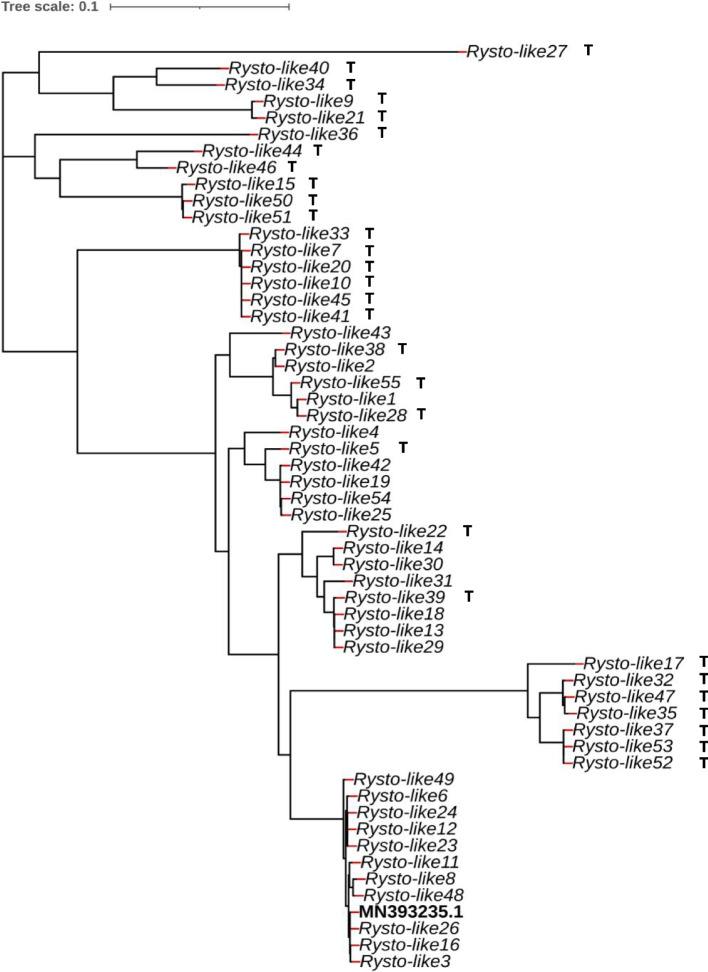
Phylogenetic tree based on the nucleotide sequences of *Rysto-like* variants detected in 10 tuber-bearing *Solanum* species and in six resistant potato controls. Thirty *Rysto-like* sequences obtained using the T primer pair (amplicons without the ATG start codon) are marked with the letter T. The phylogenetic tree was constructed using the FastTree2 tool with minimum-evolution subtree-pruning-regrafting (SPRs) and maximum-likelihood nearest-neighbour interchanges (NNIs). The length of the bar indicates 0.1 changes per nucleotide site

**Fig. 4:**
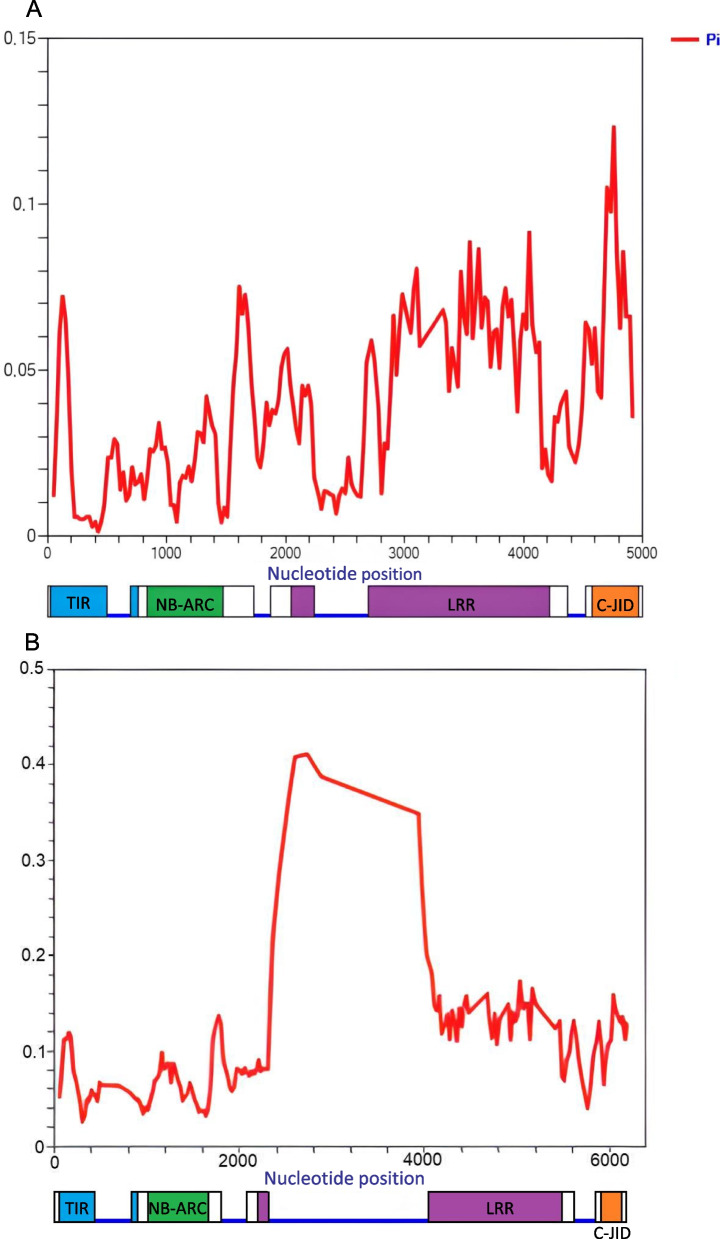
Nucleotide diversity of the *Ry*_*sto*_ homologues detected in 10 tuber-bearing *Solanum* species and six resistant potato controls. Polymorphisms are indicated by Pi values determined using DnaSP v6 (0 is conserved and 1 is maximum diversity). (A) Polymorphisms based on fragments of 25 *Rysto-like* variants obtained with primer pairs U and V. (B) Polymorphisms based on fragments of 30 *Rysto-like* variants obtained with primer pair T (amplicons without the ATG start codon). *Ry*_*sto*_ gene models under the x-axes present introns as blue lines and exons as rectangles with colour-coded domains: TIR = N-terminal domain homologous to the Drosophila Toll domain and human interleukin-1 receptor (blue), NB-ARC = nucleotide-binding domain (green), LRR = leucine-rich repeats (purple), C-JID = C-terminal jelly roll/Ig-like domain (orange). The calculations were done using a sliding window of size 100 with step size 25

A phylogenetic tree of the 55 *Rysto-like* sequences is presented in Fig. 3, and the 30 sequences obtained by primer pair T and lacking the ATG start codon are indicated. Nucleotide identity of the sequences originated from primer pairs U and V to the reference *Ry*_*sto*_ gene ranged from 93.11 to 99.98%, while that of the *Rysto-like* variants obtained from primer pair T ranged from 89.87 to 96% (Table S5). The Pi values of *Rysto-like* sequences originated from primer pairs U and V (0.003 to 0.12) was lower than that of the sequences originating from primer pair T (0.03 to 0.4) (Fig. 4 A and B). The *Rysto-like* sequences encoding the TIR domains were highly conserved in both groups. *Rysto-like* sequences encoding LRR and C-terminal jelly roll/Ig-like (C-JID) domains were highly variable, with a higher Pi value for the sequences obtained from primer pair T than that from primer pairs U and V. The third intron of the *Rysto-like* sequences obtained from primer pair T showed the greatest diversity. Insertions (from 356 to 1178 bp) and deletions (from 71 to 227 bp) were found in the third intron of 16 and eight *Rysto-like* sequences obtained from primer pair T compared to the *Ry*_*sto*_ reference gene, respectively.

### Analysis of the predicted Rysto-like protein sequences

**Fig. 5:**
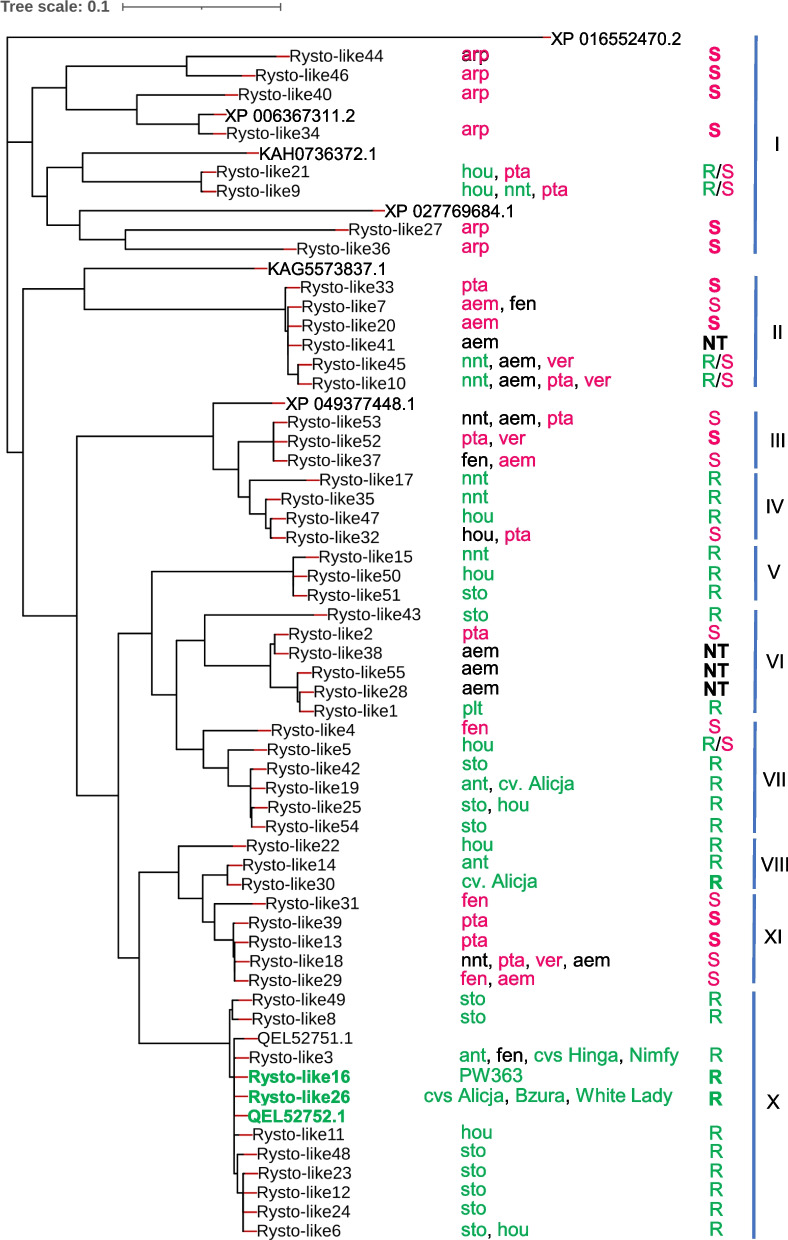
Phylogenetic tree of the predicted Ry_sto_ homologous proteins from wild relatives of potato. The predicted Rysto-like protein sequences with 100% identity to the reference Ry_sto_ protein from *S. stoloniferum* are marked in bold (green). Reference protein sequences from other species in *Solanaceae*: XP016552470.2 = disease resistance protein Roq1-like from *Capsicum annum*, XP006367311.2 = TMV resistance protein-like from *S. tuberosum*, XP027769684.1 = TMV resistance protein-like from *S. pennellii*, KAH0736372.1 = hypothetical protein from *S. tuberosum*, KAG5573837.1 = hypothetical protein from *S. commersonii*, XP049377448.1 = disease resistance protein Roq1-like from *S. stenotomum*. The QEL52751.1 sequence is a dominant isoform and QEL52752.1 sequence is a less abundant isoform of the Ry_sto_ protein from *S. stoloniferum*. The *Solanum* species in which the Ry_sto_ proteins were detected are indicated to the right of the tree. The letters S and R after the species code indicate susceptible (pink) and resistant species (green). aem = *S. aemulans*, ant = *S. antipoviczii*, arp = *S. aracc-papa*, fen = *S. fendleri*, hou = *S. hougasii*, nnt = *S. neoantipoviczii*, pta = *S. papita*, plt = *S. polytrichon*, sto = *S. stoloniferum*, ver = *S. verrucosum*. Bold indicates variants detected only in R or S genotypes. NT = not tested. The phylogenetic tree was constructed using FastTree2 tool with minimum-evolution subtree-pruning-regrafting (SPRs) and maximum-likelihood nearest-neighbor interchanges (NNIs). The length of the bar indicates 0.01 changes per amino acid site

A phylogenetic tree of the 45 unique protein sequences, which were predicted from the 55 *Rysto-like* variants, is presented in Fig. 5. We classified them into 10 groups based on the clustering and the PVY resistance. Both *Rysto-like16* (from the breeding line PW363) and *Rysto-like26* variants (from cultivars Alicja, Bzura and White Lady) encode a protein identical to the Ry_sto_ reference protein (QEL52752.1). The remaining 44 predicted Rysto-like proteins were 65.93–99.92% identical to the reference protein (Table S6). Sixteen of these 44 Rysto-like proteins were 65.93–79.93% identical to the reference protein, while they showed higher (90.24–96.52%) nucleotide identity to the *Ry*_*sto*_ reference gene (Tables S5 and S6).

**Fig. 6:**
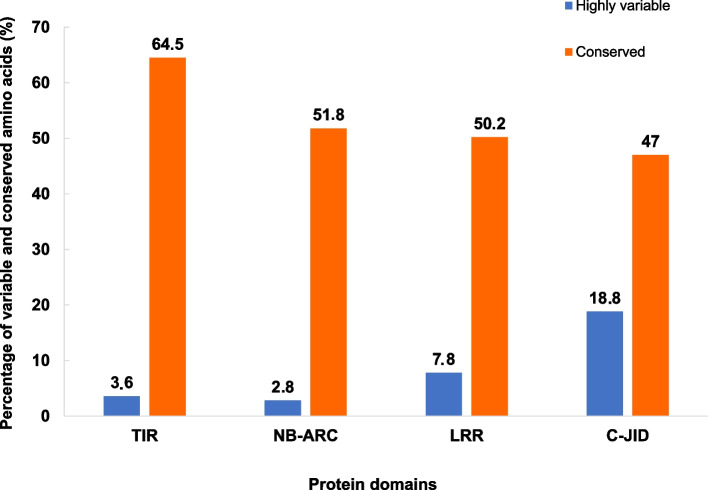
Level of amino acid conservation in the TIR, NB-ARC, LRR and C-JID domains in the predicted Rysto-like protein variants, estimated using the ConSurf algorithm. The Ry_sto_ reference protein (QEL52752.1) from *S. stoloniferum* and the predicted Rysto-like proteins from wild potato genotypes and six controls (potato cultivars Alicja, Bzura, Hinga, Nimfy, White Lady and breeding line PW363), were included in the analysis. TIR = N-terminal domain homologous to the Drosophila Toll domain and human interleukin-1 receptor, NB-ARC = nucleotide-binding domain, LRR = leucine-rich repeats, C-JID = C-terminal jelly roll/Ig-like domain

The domains TIR, NB-ARC, LRR and C-JID were predicted in all the Rysto-like proteins. TIR and NB-ARC domains showed the highest level of amino acid conservation, while LRR and C-JID domains were found to be more variable (Fig. 6). Amino acid substitutions, insertions and/or deletions were observed, especially in the LRR and C-JID domains of the predicted Rysto-like protein sequences compared to the reference protein. Single amino acid substitutions in the LRR domain (798L > V, 815E > K, 831S > I and 880S > D) and a single amino acid deletion (deletion of L at position 1228) in the C-JID domain were found in seven Rysto-like sequences from the resistant *Solanum* species that clustered with the Ry_sto_ reference protein in group X, as shown in Fig. 5, of the phylogenic tree. Single amino acid insertions in the C-JID domain were found in eight Rysto-like sequences that clustered in groups VII, VIII and IX. In case of 15 Rysto-like sequences belonging to the phylogenetic groups III, V, VI and VII, deletions of three amino acids at position 1001–1003 in the LRR domain were detected. Insertions up to 57 amino acids in the LRR domain were found in two Rysto-like sequences from the susceptible *Solanum* species that clustered in group VI. Deletions from 24 to 160 amino acids in the LRR domain were found in the 14 Rysto-like sequences clustered in groups I and II. Deletions of 20 amino acids at position 1239–1258 in the C-JID domain were observed in 13 Rysto-like sequences that belong to the phylogenetic groups II, III, IV and V.

### Assessment of *Hyoscyamus niger* and *Physalis peruviana* for presence of *Ry*_*sto*_

In both *H. niger* and *P. peruviana* (one accession of each species, four to five plants per accession), only putative fragments of *Ry*_*sto*_ gene were detected using PCR with primers that targeted the LRR, TIR or 5' UTR regions of the *Ry*_*sto*_ gene (Table S3). These fragments were sequenced by the Sanger method.

In *H. niger*, the amplicons of the TIR encoding region (amplified by primer pair I) showed 87% identity to the *Ry*_*sto*_ reference gene and 94% identity to the *N-like* TMV resistance gene from *Lycium ferocissimum*. Fragments targeting the LRR encoding region (amplified by primer pair H) showed 82% identity to the reference *Ry*_*sto*_ gene and 84% identity to the *N-like* TMV resistance gene from *S. tuberosum*. Fragments targeting the 5' UTR and LRR encoding region (amplified by primer pair J) did not show identity to any known sequences in the NCBI database (Table S7).

In *P. peruviana,* fragments targeting the LRR encoding region (amplified by primer pair K) showed 80% identity to the *Ry*_*sto*_ reference gene and 92% identity to the disease resistance gene *Roq1* from *Capsicum annuum*. Fragments targeting TIR (amplified by primer pair I) and LRR (amplified by primer pair H) encoding regions did not show any identity to the reference *Ry*_*sto*_ gene but exhibited 90% and 86% identity to the *N-like* TMV resistance gene from *S. tuberosum* and to the disease resistance gene *Roq1* from *C. annuum*, respectively (Table S7).

## Discussion

PacBio amplicon sequencing is an efficient method to study diversity of wholes gene(s). We obtained 8.35 Gb from 99 amplicons, of which 91 amplicons were *Ry*_*sto*_ homologues of ca. 5 kb and eight were homologues of a different gene of ca. 2 kb. The length of routinely obtained sequences allows to study whole genes without need for assembling. We obtained identical *Rysto-like* sequences from independently barcoded samples (cultivars Hinga and White Lady) and from samples amplified with different primer pairs (cultivar Alicja), confirming the high accuracy of PacBio SMRT CCS sequencing described previously [25, 26]. Multiple homologues and/or alleles differing by even a single nucleotide polymorphism were detected in the polyploid plants.

We identified 55 unique *Rysto-like* variants from 72 genotypes of 10 *Solanum* species and six potato controls with up to eight *Rysto-like* variants in a single genotype. In the wild relatives of potato, we detected 52 *Ry*_*sto*_ homologues demonstrating high level of variation. In contrast, only five variants were found in the potato controls, confirming that a limited number of *Ry*_*sto*_ sources was used for introgression to the cultivated potato.

### Presence of *Ry*_*sto*_ homologues in potato cultivars

The *Ry*_*sto*_ gene was detected and sequenced from the tetraploid potato cultivars Alicja, Bzura, White Lady and the breeding line PW363. Two *Ry*_*sto*_ variants were found in cultivar Alicja in addition to the variant described by Grech-Baran et al. 2020 [19], who sequenced a dihaploid clone derived from Alicja. The presence of the *Ry*_*sto*_ gene in cultivars Alicja and Bzura was also confirmed using resistance gene enrichment sequencing (RenSeq) in another laboratory (Ingo Hein, James Hutton Institute, UK, personal communication). The variant *Rysto-like26* found in cultivars Alicja, Bzura and White Lady, as well as the variant *Rysto-like16* found in the breeding line PW363, encode a protein identical to the Ry_sto_ reference protein. One of the two additional variants found in cultivar Alicja, *Rysto-like30*, showed 100% nucleotide identity to the *TMV2* gene in cultivar White Lady, identified by fine-scale genetic mapping and characterized by Sanger sequencing in a previous study [27]. *TMV2* displayed 95% identity to the reference *Ry*_*sto*_ gene published by Grech-Baran et al. 2020 [19]. Kondrák et al. (2020) concluded that it is highly probable that *TMV2* is the *Ry*_*sto*_ gene in White Lady, however, *TMV2* did not provide resistance to PVY in transgenic potato plants expressing this gene [27]. We detected a single T deletion in the poly-T homopolymeric tract in the third intron, compared to the *Ry*_*sto*_ reference gene (MN393235.1, position 5243–5260), in all tested cultivars using both PacBio and Sanger sequencing. The possible reason might be different plant material used in this study (the tetraploid potato cultivar Alicja) and the previous study (the dihaploid clone Alicja) [19] or just sequencing errors, as such regions are prone to errors.

Different *Rysto-like* variants identified in different cultivars tested, as well as additional variants identified in cultivar Alicja in this study, may reflect different origins of the gene [15, 19, 27]. The source of the *Ry*_*sto*_ reference gene is the dihaploid clone Alicja, which was obtained from the tetraploid potato cultivar Alicja via parthenogenesis [19, 28]. Reduction of ploidy may have led to loss of the *Ry*_*sto*_ variants, which we detected in the tetraploid cultivar Alicja. The source of *Ry*_*sto*_ in PW363, called *Ry-f*_*sto*_ by Flis et al. 2005 [15], is different than that of the other potato cultivars tested in this study, which was confirmed by finding that the *Rysto-like16* sequence present only in this breeding line differed by a single nucleotide from *Rysto-like26* found in cv Alicja. In contrast, Grech-Baran et al. (2020) identified a variant identical to the *Ry*_*sto*_ reference gene in PW363, using a different primer pair [19]. Cultivars Nimfy and Hinga both contained *Rysto-like3*, confirming their common ancestor stoXIIB, which is considered the source of the *Ry*_*sto*_ gene in these cultivars [18]. Alicja and Bzura both contained *Rysto-like26*, confirming the common ancestor MPI 55.957/54 [18, 19]. Cultivar White Lady also contained *Rysto-like26*, but the exact *S. stoloniferum* source of this cultivar is not known [29].

### Potential new sources of PVY resistance in tuber-bearing *Solanum* species

We provide evidence of PVY resistance in four *Solanum* species including *S. antipoviczii* and *S. hougasii*. *S. antipoviczii*, four genotypes of *S. hougasii*, as well as *S. neoantipoviczii* exhibited resistance to multiple strains of PVY, while *S. polytrichon* showed resistance to one PVY strain. The *S. stoloniferum* accession POL003:333100 was not tested for resistance to PVY in this study, but it has been tested and showed resistance to three PVY strains in a previous study [8]. PVY resistance in *S. neoantipoviczii* was consistent with previous results [8]. For *S. polytrichon*, resistance to PVY^NTN^ was demonstrated in the two tested genotypes, whereas Zoteyeva et al. (2012) showed resistance to PVY^O^ and PVY^N−Wi^ and in contrast susceptibility to PVY^NTN^ using the same accessions as in this study [8]. The segregation of resistance and small number of tested plant genotypes for this particular accession could be the reasons of this discrepancy.

Several genotypes of *S. antipoviczii*, *S. hougasii* and *S. neoantipoviczii*, in which resistance to PVY was demonstrated in this study, could be potential sources of new genes for resistance to PVY. However, we were not able to amplify the full coding regions of the *Ry*_*sto*_ homologues in these genotypes, only fragments of TIR and LRR region were detected. Although *S. dolichostigma* (POL333114, k-7610) and *S. gibberulosum* (POL333103, k-2937) were shown to exhibit PVY resistance in a previous study [8], neither full coding *Ry*_*sto*_ regions nor fragments were detected in this study. Overall, some of the *Rysto-like* variants identified in this study differed from the *Ry*_*sto*_ reference gene and could therefore be potential resistance gene sources against PVY and other potyviruses in potato and other crops.

### Inter-species and inter-genotype variability of *Rysto-like* variants among the wild relatives of potato

Of the 26 tested tuber-bearing *Solanum* species in this study, *Ry*_*sto*_ homologues were identified in *S. stoloniferum* and six other species from Mexico, *S. aemulans* from Argentina, and two accessions of unknown origin. Among the *Solanum* species in which *Ry*_*sto*_ variants were detected, five species as defined by Hawkes 1990 [30], *S. antipoviczii*, *S. fendleri*, *S. neoantipoviczii*, *S. papita* and *S. polytrichon*, are classified as *S. stoloniferum* according to Spooner et al. 2016 [31]. The distribution of the *Rysto-like* variants supports the close relationship of those species and Spooner’s taxonomy. These five species and *S. stoloniferum* are tetraploid, while *S. hougasii*, *S. aemulans* and *S. verrucosum* are hexaploid, triploid and diploid, respectively. *S. aracc-papa* has unknown ploidy level. Irrespective of ploidy level, from one to eight *Rysto-like* variants were detected per genotype, indicating that we amplified not only the alleles of the gene but also similar sequences possibly from other loci.

Inter-species and inter-genotype variability of the *Rysto-like* variants were observed in the studied genus *Solanum*. The variants clustered in different phylogenic groups. Among the five resistant species, the number of *Rysto-like* variants found in *S. stoloniferum*, *S. hougasii*, *S. neoantipoviczii* were higher than those found in *S. polytrichon* and *S. antipoviczii*. In *S. stoloniferum*, 12 variants (two to eight per genotype) were identified, showing the presence or absence of polymorphism between genotypes in the tested accession of this species.

We demonstrated high level sequence variation in LRR and C-JID domains of the predicted Rysto-like immune receptors that might indicate different pathogen or strain recognition spectra of some of the Rysto-like proteins. Both LRR and C-JID (also called post-LRR) domains are involved in effector recognition [32]. The deletions and insertions were detected in both domains of the receptor especially in the susceptible *Solanum* species. Pathogen effectors interact with the LRR and C-JID domains leading to oligomerization of the receptor and finally formation of tetrameric TIR-NB-LRR (TNL) resistosomes [33, 34]. Mutations in the C-JID domain may hinder TNL-mediated resistance [35].

### Hyoscyamus niger and Physalis peruviana

This study identified only short fragments amplified by the *Ry*_*sto*_-specific primers in *H. niger* and *P. peruviana*. However, these fragments showed a higher level of identity to *N-like* TMV resistance gene and disease resistance gene *Roq1* than to the *Ry*_*sto*_ reference gene. The lower nucleotide sequence identity to the *Ry*_*sto*_ gene might be the reason for the failure of amplification of the full length of this gene in these two species. We further demonstrated that *H. niger* is a host for PVY and that the PVY strains derived from potato can infect *P. peruviana*. PVY can in principle impair *H. niger* and *P. peruviana* cultivation in the region where these two species are grown. Severe symptoms induced by PVY infection in *H. niger* observed here, may cause yield reduction. Moreover, both species could be reservoirs for PVY in nature, impacting potato production and other *Solanaceae* crops. In contrast to our findings, Green et al. (2017) could not show that PVY isolates from *P. peruviana* were able to infect potato [36]. Although *P. peruviana* showed strain-specific resistance to the virus, the tested accession of *H. niger* was susceptible to all PVY strains used in this study. Search for resistance among different *H. niger* and *P. peruviana* accessions or use of *Ry*_*sto*_ might be options for obtaining resistant cultivars in these two species.

## Conclusions

This study described diversity of the PVY resistance gene *Ry*_*sto*_ in potato and its wild relatives. We obtained 53 new variants of the *Ry*_*sto*_ gene in *Solanum* species from Mexico or Argentina. Higher levels of diversity of the *Rysto-like* sequences were found in the wild relatives of potato than in the resistant control potato cultivars. Several *Solanum* species, including *S. antipoviczii* and *S. hougasii*, showed resistance to PVY. The new *Rysto-like* variants and the identified PVY resistant potato genotypes are potential resistance sources against PVY in potato breeding. Moreover, this study demonstrated that *H. niger* is a new host of PVY and that *P. peruviana* shows PVY strain-specific resistance, which is significant finding for cultivation of these species, as well as for general PVY management and ecology. The amplicon sequencing based on PacBio SMRT and the following data analysis pipeline described in our work may be applied to obtain the nucleotide sequences and analyse any full-length genes from any, even polyploid, organisms.

## Materials and methods

### Plant materials

In total, 26 tuber-bearing *Solanum* species (29 accessions, 298 genotypes) from the National Centre for Plant Genetic Resources: Polish Genebank (IHAR-PIB, Radzików, Poland) were used (Table S2). We also included *H. niger* (accession TN-82–763) and *P. peruviana* (accession TN-82–765) from the National Plant Gene Bank of Iran (Mahdasht Road, Karaj, 31359–33151, Iran). Potato cultivars Alicja, Bzura, Hinga, Nimfy, White Lady and the potato breeding line PW363 known to carry the *Ry*_*sto*_ gene, were used as resistant controls, while cultivars Irys, Irga, Nicola and Vineta without the *Ry*_*sto*_ gene were used as susceptible controls [15, 19, 27].

### DNA extraction and amplification of *Ry*_*sto*_

Genomic DNA was extracted from young leaves of plants growing in a greenhouse using the DNeasy Plant Mini kit (Qiagen, Hilden, Germany) according to the manufacturer’s instructions. All primers used in this study are listed in Table S8. Primers for the detection of *Ry*_*sto*_ gene fragments (pairs A, C, I, J, H and K) and amplification of fragments spanning the complete coding region including introns (pairs T and U) were designed based on the *S. stoloniferum Ry*_*sto*_ gene sequence (MN393235.1) using Primer-BLAST [37]. The *Ry*_*sto*_ specific primer pair 630-35S-F/630-35S-R described by Grech-Baran et al. 2020 [19], which was named primer pair V in this study, was also used for amplifying the *Ry*_*sto*_ full coding region. Primer pair RystoF2/R3 for amplification of a fragment containing the third intron of the *Ry*_*sto*_ gene was designed manually. The short fragments and fragments containing the full coding region of the *Ry*_*sto*_ gene were amplified using DreamTaq DNA Polymerase and Phusion™ High–Fidelity DNA Polymerase (both from Thermo Fisher Scientific Inc., Waltham, MA, USA), respectively. The PCR reaction mixture and conditions are shown in Table S9. In each reaction, 1 µL of genomic DNA (concentration ranged from 11 ng/μL to 110 ng/μL) was used as a template. PCR products were analysed using electrophoresis in 1.5% agarose gels or in 1% agarose gel (for fragments with full coding region).

### Sample preparation for PacBio sequencing

The *Ry*_*sto*_ gene homologues were amplified with barcoded primer pairs dedicated to each potato genotype. Amplicons obtained with primer pair U from the resistant control cultivars Hinga and White Lady were barcoded in two replicates with two independent barcodes for each cultivar. Amplicons obtained with both V and U primer pairs from cultivar Alicja were also barcoded independently. Barcodes of 16 bp in length were chosen from a list available on the PacBio website (https://www.pacb.com/). The barcoded T, U, and V primers are listed in Table S10. The barcoded PCR products were cleaned from nucleotides, primers, buffer and non-specific products below 1000 bp with magnetic beads using the AMPure XP kit (Beckman Coulter Inc., Brea, CA, USA) according to the manufacturer’s instructions. Amplicons obtained from one to three potato genotypes were cleaned together. The concentrations and lengths of the obtained products were measured using a 2100 Bioanalyzer and Agilent DNA 7500 Reagents kit (Agilent Technologies Inc. Santa Clara, CA, USA). Purified PCR products were mixed in an equimolar ratio and sequenced by the PacBio SMRT CCS method in a single SMRT Cell 8 M (Sequel II system) at the Norwegian Sequencing Centre (https://www.sequencing.uio.no/).

### PacBio sequence data analysis

CCS sequences were generated using the CCS pipeline (SMRT Link v10.1.0.119588) with default settings (minimum number of passes 3, minimum predicted accuracy 0.99) at the Norwegian Sequencing Centre. Barcode identification and sample demultiplexing were performed using the Lima tool (version 2.4.0; https://lima.how/). DADA2, an open-source R package, was used to designate amplicon sequence variants from the HiFi reads [38]. The obtained unique *Ry*_*sto*_ homologous sequences were annotated using FGENSH (http://www.softberry.com/berry.phtml). The nucleotide sequences of *Ry*_*sto*_ gene variants, as well as the predicted Ry_sto_ homologous protein sequences, were aligned using ClustalW (https://www.genome.jp/tools-bin/clustalw). Phylogenetic trees were constructed using FastTree2 with minimum-evolution subtree-pruning-regrafting (SPRs) and maximum-likelihood nearest-neighbour interchanges (NNIs). All trees were visualized by iTOL online tool (https://itol.embl.de/). Nucleotide diversity (Pi) of *Ry*_*sto*_ homologues from *Solanum* species was analysed using DnaSP v6 [39]. InterPro was used for functional analysis of the predicted protein variants by domain prediction [40]. The degree of domain conservation in the predicted Ry_sto_ protein variants was analysed using ConSurf on-line tool [41].

### Sanger sequencing and data analysis

*Ry*_*sto*_ gene fragments were obtained with primer pairs H, I and K for *P. peruviana*, H, I and J for *H. niger*, and RystoF2/R3 for the potato breeding line PW363 and cultivars Alicja, Bzura, White Lady, Hinga and Nimfy. PCR products were purified with GenElute PCR Clean-Up kit (Sigma-Aldrich, Merck, Germany) according to the manufacturer's protocol, and sequenced by the Institute of Biochemistry and Biophysics, Polish Academy of Sciences, Warsaw, Poland, or Genomed S.A., Warsaw, Poland. The resulting sequences were analysed using Chromas 2.6.6 (https://technelysium.com.au/wp/chromas/).

### PVY resistance test

Resistance of nine tuber-bearing *Solanum* species (11 accessions, 52 genotypes, 1246 plants), *H. niger* (accession TN-82–763, 54 plants), and *P. peruviana* (accession TN-82–765, 79 plants) to PVY was tested. The nine *Solanum* species were *S. aemulans*, *S. antipoviczii*, *S. aracc-papa*, *S. fendleri*, *S. hougasii*, *S. neoantipoviczii*, *S. papita*, *S. polytrichon* and *S. verrucosum*. Potato cultivars Bzura, Hinga and White Lady were used as resistant controls, and cultivars Irga and Irys as susceptible controls. PVY resistance tests were conducted in two subsequent growing seasons, 2021 and 2022, in a greenhouse (20–28°C, natural light) between May and September. Briefly, the lower two leaves of potato plants at 5–6 leaf stage were lightly sprinkled with carborundum powder. Systemically infected tobacco leaves were ground in sterile water with a volume ratio of 1:20 and used as inoculum. Mechanical inoculation was conducted by applying the inoculum to the prepared lower two leaves [42]. Five PVY strains were used: PVY^NTN^ (isolate PVY-3202, accession KX356068.1), PVY^N−Wi^ (isolate PVY-3411, accession KX356069.1), PVY^O^ (isolate PVY-19Raj09, accession OP643837), PVY^E^ (isolate PVY-27Vin09, accession OP643836) and PVY^Z^-NTN (isolate PVY-3303, accession KX356070.1). For the tested *Solanum* species, the tuber-propagated plants were tested for the presence of PVY, potato virus M, potato leafroll virus, potato virus S and potato virus X by ELISA. Only tubers derived from plants negative in ELISA were used for the PVY resistance test. Two to eight genotypes were tested per accession and two to 90 plants were tested per genotype. The genotypes with 10 plants or less were inoculated with PVY^NTN^ strain, those with 20 plants were inoculated with PVY^NTN^ and PVY^N−Wi^ strains, and the ones with more than 50 plants were inoculated with each of the five strains. For each PVY strain, two to 21 plants per genotype were inoculated. *H. niger* and *P. peruviana* plants were inoculated with each of the five PVY strains. For each strain, 10 to 16 plants per accession were inoculated. The presence of PVY in the upper non-inoculated leaves was tested by ELISA [43] using monoclonal cocktail antibody (112911, BIOREBA, Switzerland) that recognizes all strains of PVY. For *Solanum* species and *P. peruviana*, the ELISA were conducted between 25 to 35 dpi, and the samples showing negative reaction were tested again at 48–66 and 75–90 dpi. For *H. niger*, ELISA were conducted at 14 dpi.

### Supplementary Information

Additional file 1: Table S1. Resistance of tuber-bearing *Solanum* species, *Hyoscyamus niger* and *Physalis peruviana* accessions to potato virus Y; Table S2. Tuber-bearing *Solanum* species used to search for* Ry*_*sto*_ gene homologues; Table S4. PacBio amplicon sequencing of *Ry*_*sto*_ coding region; Table S7. Analysis of the fragments of *Ry*_*sto*_ homologues in *Hyoscyamus niger* and *Physalis peruviana* obtained by Sanger sequencing; Table S8. Primers for amplification of *Ry*_*sto*_ homologues in *Solanum* species, *Hyoscyamus niger* and *Physalis peruviana*; Table S9. PCR reaction mixture and conditions; Table S10. *Ry*_*sto*_-specific primer pairs V, U and T with PacBio barcodes.

Additional file 2: Table S3. Detection of *Ry*_*sto*_ homologues in selected tuber-bearing *Solanum* species, *Hyoscyamus niger*, *Physalis peruviana* and results of PVY resistance test.

Additional file 3: Table S5. Information about sequenced variants of the *Ry*_*sto*_ gene in wild potato species and resistant controls.

Additional file 4: Table S6. Comparison of protein sequences of the obtained variants of the Ry_sto_ protein (identity %).

Additional file 5: Figure S1. Screening for the presence of *Ry*_*sto*_ homologues in wild relatives of potato. In total, 298 potato genotypes representing 29 accessions of 26 tuber-bearing *Solanum* species were examined. Six resistant control genotypes, potato cultivars Alicja, Bzura, Hinga, Nimfy, White Lady and the breeding line PW363, were included. (A) Number of potato genotypes in which fragments of the *Ry*_*sto*_ gene were detected using primer pairs A, C and J. (B) Number of potato genotypes in which the full coding sequence of the *Ry*_*sto*_ gene were detected using primer pairs T, U and V. (C) Number of potato genotypes in which the *Ry*_*sto*_ homologues were obtained using the barcoded primer pairs T, U and V. (D) A schematic view of the *Ry*_*sto*_ gene, indicating the different target regions amplified by primer pairs A, C, J, T, U and V. Exons are shown as rectangles with colour-coded domains: UTR = untranslated region, TIR = N-terminal domain homologous to the Drosophila Toll domain and human interleukin-1 receptor, NB-ARC = nucleotide-binding domain, LRR = leucine-rich repeat motif, C-JID = C-terminal jelly roll/Ig-like domain.

Additional file 6: Figure S2. Pedigrees of the resistant potato controls. (A) Alicja. (B) Bzura. (C) White Lady. (D) Nimfy. (E) Hinga. (F) breeding line PW363. The year of registration of the cultivars and level of resistance to potato virus Y (PVY) are given in brackets (R = resistant, M = medium resistant). The likely source of PVY resistance is in bold. StoXIIB is the name of a *S. stoloniferum* clone from Plant Breeding and Acclimatization Institute – National Research Institute, Poland.

## Data Availability

All data supporting the findings of this study are available within the paper and its Supplementary Information. The 55 unique *Rysto-like* sequences have been deposited to the NCBI GenBank with the accession numbers from PP061793 to PP061847. The raw data of the PacBio sequencing results have been deposited in the NCBI Sequence Read Archive (SRA) (accession number SRR27478595, https://www.ncbi.nlm.nih.gov/sra/?term=SRR27478595). Code used to process the raw data with Lima and DADA2 and intermediary files can be found in the GitLab repository https://gitlab.nibio.no/simeon/rysto-supplementary-code. The plant materials used in this study are reserved in the National Centre for Plant Genetic Resources: Polish Genebank (IHAR-PIB, Radzików, Poland, https://bankgenow.edu.pl/en/) and the National Plant Gene Bank of Iran (Mahdasht Road, Karaj, 31359-33151, Iran, https://medomed.org/featured_item/national-plant-gene-bank-of-iran/), and are available on request.
