# Supplementary material for: Diversity of the Rysto gene conferring resistance to potato virus Y in wild relatives of potato
Source: BMC Plant Biol. 2024 May 8;24:375. doi: 10.1186/s12870-024-05089-2 (PMC11077776; doi:10.1186/s12870-024-05089-2)
Supplement: Supplementary file 1 — Additional file 1: Table S1. Resistance of tuber-bearing Solanum species, Hyoscyamus niger and Physalis peruviana accessions to potato virus Y; Table S2. Tuber-bearing Solanum species used to search for Rysto gene homologues; Table S4. PacBio amplicon sequencing of Rysto coding region; Table S7. Analysis of the fragments of Rysto homologues in Hyoscyamus niger and Physalis peruviana obtained by Sanger sequencing; Table S8. Primers for amplification of Rysto homologues in Solanum species, Hyoscyamus niger and Physalis peruviana; Table S9. PCR reaction mixture and conditions; Table S10. Rysto-specific primer pairs V, U and T with PacBio barcodes. [file 12870_2024_5089_MOESM1_ESM.docx]

**Table S1.** Resistance of tuber-bearing Solanum species, Hyoscyamus niger and Physalis peruviana accessions to potato virus Y

| *Solanum* species | Accession number POL003 | Genotypes | Number of PVY-positive plants / Number of total tested plants | | | | |
| --- | --- | --- | --- | --- | --- | --- | --- |
|  |  |  | PVY strains | | | | |
|  |  |  | PVY^NTN^ | PVY^N-Wi^ | PVY^O^ | PVY^E^ | PVY^Z^-NTN |
| *S. aemulans* | 333119 | 17-28/1 | 4/4 | - | - | - | - |
|  |  | 17-28/5 | 7/10 | 10/10 | - | - | - |
| *S. antipovichii* | 333099 | 17-24/2 | **0/7** | - | - | - | - |
|  |  | 17-24/3 | **0/11** | **0/5** | - | - | - |
|  |  | 17-24/6 | **0/2** | - | - | - | - |
|  |  | 17-24/7 | **0/10** | **0/6** | - | - | - |
|  |  | 17-24/9 | **0/9** | - | - | - | - |
| *S. aracc-papa* | 333150 | 17-35/5 | 10/10 | 10/10 | 11/11 | 9/9 | - |
|  |  | 17-35/8 | 19/19 | 19/19 | 19/19 | 13/13 | 11/11 |
| *S. fendleri* | 333110 | 17-25/3 | 10/10 | 10/10 | 10/10 | 17/17 | 10/10 |
|  |  | 17-25/4 | 10/10 | 4/4 | - | - | - |
|  |  | 17-25/5 | 10/10 | 10/10 | 9/9 | 10/10 | 9/9 |
|  |  | 17-25/7 | 20/20 | 10/10 | 11/11 | 10/10 | 13/13 |
| *S. fendleri* | 333112 | 17-26/5 | 21/21 | 13/13 | 12/12 | 2/2 | 8/8 |
|  |  | 17-26/8 | 12/12 | 12/12 | 12/12 | 7/7 | 10/10 |
|  |  | 17-26/9 | 16/16 | 16/16 | 16/16 | 17/17 | 16/16 |
|  |  | 17-26/10 | 10/10 | 10/10 | 12/12 | - | - |
| *S. hougasii* | 333148 | 16-42/2 | **0/3** | - | - | - | - |
|  |  | 16-42/18 | 9/9 | - | - | - | - |
|  |  | 16-42/20 | **0/10** | **0/9** | - | - | - |
|  |  | 16-42/21 | **0/9** | **0/10** | **0/3** | - | - |
|  |  | 16-42/22 | **0/13** | **0/10** | - | - | - |
| *S. neoantipoviczii* | 333117 | 17-27/1 | **0/7** | - | - | - | - |
|  |  | 17-27/2 | **0/11** | - | - | - | - |
|  |  | 17-27/3 | **0/2** | - | - | - | - |
|  |  | 17-27/4 | **0/10** | **0/10** | **0/10** | **0/2** | - |
|  |  | 17-27/5 | **0/19** | **0/10** | **0/4** | - | - |
|  |  | 17-27/6 | **0/10** | **0/7** | - | - | - |
| *S. papita* | 333147 | 16-41/1 | 10/10 | 11/11 | 9/9 | - | - |
|  |  | 16-41/4 | 10/10 | 9/9 | - | - | - |
|  |  | 16-41/8 | 10/10 | 10/10 | 1/1 | - | - |
|  |  | 16-41/9 | 10/10 | 11/11 | 4/4 | - | - |
|  |  | 16-41/10 | 10/10 | 8/8 | - | - | - |
|  |  | 16-41/11 | 10/10 | 10/10 | 12/12 | 2/2 | - |
|  |  | 16-41/12 | 10/10 | 11/11 | - | - | - |
|  |  | 16-41/13 | 18/18 | 17/17 | 17/17 | 20/20 | 18/18 |
| *S. papita* | 333159 | 17-37/3 | 10/10 | 10/10 | 10/10 | 5/5 | - |
|  |  | 17-37/4 | 10/10 | 5/5 | - | - | - |
|  |  | 17-37/5 | 10/10 | 10/10 | 10/10 | 10/10 | 4/4 |
|  |  | 17-37/8 | 10/10 | 2/2 | - | - | - |
|  |  | 17-37/10 | 5/5 | - | - | - | - |
|  |  | 17-37/12 | 10/10 | 1/1 | - | - | - |
|  |  | 17-37/27 | 7/7 | - | - | - | - |
| *S. polytrichon* | 333108 | 16-36/1 | **0/6** | - | - | - | - |
|  |  | 16-36/10 | **0/3** | - | - | - | - |
| *S. verrucosum* | 333157 | 17-36/1 | 10/10 | 4/4 | - | - | - |
|  |  | 17-36/6 | 7/7 | - | - | - | - |
|  |  | 17-36/7 | 10/10 | 10/10 | 2/2 | - | - |
|  |  | 17-36/8 | 7/7 | - | - | - | - |
|  |  | 17-36/9 | 10/10 | - | - | - | - |
|  |  | 17-36/12 | 7/7 | - | - | - | - |
|  |  | 17-36/15 | 8/8 | - | - | - | - |
|  |  |  |  |  |  |  |  |
| *Hyoscyamus*  *niger* | IRN:TN-82-763 |  | 11/11 | 10/10 | 11/11 | 11/11 | 11/11 |
| *Physalis peruviana* | IRN:TN-82-765 |  | 10/15 | 15/16 | **0/16** | 8/16 | 10/16 |
|  |  |  |  |  |  |  |  |
| Potato controls |  | Bzura | **0/10** | **0/10** | **0/10** | **0/10** | **0/10** |
|  |  | Hinga | **0/10** | **0/10** | **0/10** | **0/10** | **0/10** |
|  |  | White Lady | **0/11** | **0/5** | **0/5** | **0/8** | **0/2** |
|  |  | Irga | 10/10 | 10/10 | 10/10 | 10/10 | 9/9 |
|  |  | Irys | 10/10 | 10/10 | 10/10 | 10/10 | 9/9 |

*Solanum* species classification was according to Hawkes (1990) [30]; *S. fendleri*, *S .papita* and *S. polytrichon* were classified as *S. stoloniferum* according to Spooner et al. (2016) [31]. POL003 = accession number in the National Centre for Plant Genetic Resources: Polish Gene Bank (IHAR-PIB, Radzików, Poland), IRN (TN-82-763 and TN-82-765) = accession number of Plant Genetics and Genetic Resources (The National Plant Gene Bank of Iran). Resistance to PVY was measured by the lack of detected PVY using ELISA. “-” indicates not tested plants. Bold indicates PVY not detected

**Table S2.** Tuber-bearing *Solanum* species used to search for *Ry_sto_* gene homologues

| Species according to | | Accession No. (POL003) | No. of genotypes | Ploidy | EBN | Accession origin | Species distribution |
| --- | --- | --- | --- | --- | --- | --- | --- |
| Hawekes 1990 | Spooner et al. 2016 |  |  |  |  |  |  |
| *S. acaule* | *S. acaule* Bitter | 333155 | 10 | 4x | 2 | BOL | ECU, PER, BOL, ARG, CHL |
| *S. aemulans* | *S. aemulans* Bitter & Wittm | 333119 | 11 | 3x | 2 | ARG | ARG |
| *S. albicans* | *S. albicans* Ochoa | 333125 | 10 | 6x | 4 | BOL | ECU, PER |
| *S. antipoviczii* | *S. stoloniferum* Schltfl. & Bouche | 333099 | 10 | 4x | 2 | MEX | USA, MEX |
| *S. aracc-papa* | - | 333150 | 10 | - | - | - | - |
| *S. berthaultii* | *S. berthaultii* Hawkes | 333129 | 10 | 2x | 2 | BOL | BOL, ARG |
| *S. dolichostigma* | *S. chacoense* Bitter | 333114 | 10 | 2x | - | ARG | - |
| *S. famatinae* | *S. chacoense* Bitter | 333139 | 12 | 2x | - | ARG | BOL, ARG |
| *S. fendleri* | *S. stoloniferum* Schltfl. & Bouche | 333110 | 11 | 4x | 2 | MEX | USA, MEX |
| *S. fendleri* | *S. stoloniferum* Schltfl. & Bouche | 333112 | 11 | 4x | 2 | MEX | USA, MEX |
| *S. gibberulosum* | *S. chacoense* Bitter | 333103 | 10 | 2x | - | ARG | PER, ARG, PRY, URY, BRA |
| *S. guerreroense* | *S. guerreroense* Correll | 333096 | 10 | 6x | 4 | MEX | MEX |
| *S. hougasii* | *S. hougasii* Correll | 333148 | 10 | 6x | 4 | MEX | MEX |
| *S. kurtzianum* | *S. kurtzianum* Bitter & Wittm | 333121 | 12 | 2x | 2 | - | ARG |
| *S. kurtzianum* | *S. kurtzianum* Bitter & Wittm | 333130 | 1 | 2x | 2 | - | ARG |
| *S. leptophyes* | *S. brevicaule* Bitter | 333113 | 10 | 2x | - | BOL | BOL, ARG |
| *S. microdontum* | *S. microdontum* Bitter | 333149 | 13 | 2x | 2 | - | BOL, ARG |
| *S. neoantipoviczii* | *S. stoloniferum* Schltfl. & Bouche | 333117 | 10 | 4x | 2 | MEX | USA, MEX |
| *S. papita* | *S. stoloniferum* Schltfl. & Bouche | 333147 | 10 | 4x | 2 | MEX | USA, MEX |
| *S. papita* | *S. stoloniferum* Schltfl. & Bouche | 333159 | 12 | 4x | 2 | MEX | USA, MEX |
| *S. parodii* | *S. chacoense* Bitter | 333069 | 10 | 2x | - | ARG | PER, ARG, PYR, URY, BRA |
| *S. polytrichon* | *S. stoloniferum* Schltfl. & Bouche | 333108 | 10 | 4x | 2 | MEX | USA, MEX |
| *S. punae* | *S. acaule* Bitter | 333138 | 10 | 4x | 2 | PER | ECU, PER, BOL, ARG |
| *S. ruiz-ceballosii* | *S. brevicaule* Bitter | 333074 | 10 | 2x | 2 | BOL | BOL, ARG |
| *S. simplicifolium* | *S. microdontum* Bitter | 333141 | 12 | 2x | 2 | ARG | BOL, ARG |
| *S. sparsipilum* | *S. brevicaule* Bitter | 333124 | 10 | 2x | - | BOL | - |
| *S. stoloniferum* | *S. stoloniferum* Schltfl. & Bouche | 333100 | 10 | 4x | 2 | MEX | USA, MEX |
| *S. ujunense* | *S. acaule* Bitter | 333071 | 10 | 4x | 2 | BOL | BOL |
| *S. verrucosum* | *S. verrucosum* Schldl. | 333157 | 13 | 2x | 2 | - | MEX |

Accession number in the National Centre for Plant Genetic Resources: Polish Gene Bank (IHAR-PIB, Radzików, Poland). No. = Number, EBN = Endosperm Balance Number,^”^“-” = No data

**Table S4.** PacBio amplicon sequencing of Ry_sto_ coding region

| Results |  |
| --- | --- |
| Number of HiFi reads | 1 761 425 |
| HiFi yield | 8.35 Gb |
| HiFi read length (mean, bp) | 4 741 bp |
| HiFi read quality (median) | Q39 |
| HiFi number of passes (mean) | 18 |

PacBio amplicon sequencing was conducted at Norwegian Sequencing Centre (<https://www.sequencing.uio.no/>).

Circular consensus sequences (CCS) were generated using CCS pipeline (SMRT Link v10.1.0.119588) and default settings (minimum number of passes 3, minimum predicted accuracy 0.99). Data were obtained from 99 amplicons, of which 91 amplicons were *Ry_sto_* homologues of ca. 5 kb and eight were homologues of a different gene of ca. 2 kb

| Species | No. of plants | PCR primer pair | Sequenced product length (bp) | Nucleotide identity (%) to the reference gene *Ry_sto_* (MN393235.1) | Maximum nucleotide identity (%) to other genes | | | |
| --- | --- | --- | --- | --- | --- | --- | --- | --- |
|  |  |  |  |  | Species | Name | Nucleotide identity (%) | Accession |
| *H. niger* | 3 | I | 580 | 86.96 | *L. ferocissimum* | Predicted TMV resistance gene *N-like* | 93,89 | XM_059429778.1 |
|  | 2 | J | 614 | NF | NF | NF | NF | NF |
|  | 3 | H | 743 | 81.66 | *S. tuberosum* | Predicted TMV resistance gene *N-like* | 83.83 | XM_015306548.1 |
| *P. peruviana* | 3 | I | 472 | NF | *S. tuberosum* | Predicted TMV resistance gene *N-like* | 92.31 | XM_006367249.2 |
|  | 3 | H | 757 | NF | *C. annuum* | Predicted disease resistance gene *Roq1-like* | 86.05. | XR_007048489.1 |
|  | 2 | K | 507 | 80.23 | *C. annuum* | Predicted disease resistance gene *Roq1* | 91.58 | XM_016696926.2 |

**Table S7.** Analysis of the fragments of Ry_sto_ homologues in Hyoscyamus niger and Physalis peruviana obtained by Sanger sequencing

Nucleotide identity was determined based on data from NCBI nucleotide BLASTn. No. = Number, NF = Not found

**Table S8.** Primers for amplification of *Ry_sto_* homologues in *Solanum* species, *Hyoscyamus niger* and *Physalis peruviana*

| Primer name | Primer | Sequence  (5' to 3') | Position | Target region | Expected product length (bp) | T_A_ (°C) |
| --- | --- | --- | --- | --- | --- | --- |
| **Primers for amplification of short *Ry_sto_* gene fragments** | | | | | | |
| A | F | ATGCTGCAGGTGCAACAATG | 582-601 | 5'UTR | 830 | 60 |
|  | R | CTGACCTGGGAGAACCACAC | 1392-1411 | 5'UTR |  |  |
| C | F | TTTCCGGATTAGCTACCGCC | 5137-5156 | Intron | 1399 | 60 |
|  | R | GGTAGCTCTCCGGGCAATTT | 6516-6535 | coding (LRR) |  |  |
| H | F | CCCTCTCTTGGACAGTGCAG | 5397-5416 | coding (LRR) | 831 | 60 |
|  | R | AGGTGGTTGGGAGATTGCAG | 6208-6227 | coding (LRR) |  |  |
| I | F | ACGCCATCAAAATCCCCCTT | 2856-2875 | coding (TIR) | 584 | 60 |
|  | R | TTTCTGTTTTGCCGATGCCG | 3420-3439 | coding |  |  |
| J | F | TTGTTGGTGGGGCCTTTCAT | 2371-2390 | 5'UTR | 505 | 60 |
|  | R | AAGGGGGATTTTGATGGCGT | 2856-2875 | coding (TIR) |  |  |
| K | F | CCATCTCTCAGCGGCCTTAG | 5607-5626 | coding (LRR) | 630 | 60 |
|  | R | GATGGAGGAAGGTGGTTGGG | 6217-6237 | coding (LRR) |  |  |
| **Primers for amplification of fragments containing the complete coding sequence of *Ry_sto_*** | | | | | | |
| T | F | TCTCATGCTTCTTCTTCCAAAGT | 2521-2543 | coding | 4772 | 64 |
|  | R | ACCTTTGCATCACTCATTGCTT | 7271-7292 | 3'UTR |  |  |
| U | F | TGTTGCAGACAGAGTGTGGA | 2420-2439 | 5'UTR | 4942 | 64 |
|  | R | CGTGTTTGGTTGAGTACGGA | 7342-7361 | 3'UTR |  |  |
| V | F | GGTGCTAAGAAGACTTCATATCAG | 2465-2488 | 5'UTR | 5065 | 62 |
|  | R | GGATACTCAACGTATCTACCTTATTATAA | 7502-7529 | 3'UTR |  |  |
| **Primers for amplification of fragments containing the third intron of the *Ry_sto_* gene includining the poly-T homopolymeric tract** | | | | | | |
| Rysto2 | F | CTATTAGGAGTACTACTTAAGCTC | 5213-5236 | Intron III | 372 | 55 |
| Rysto3 | R | GGATCCTACATCGAGCGTC | 5567-5585 | coding (exon IV) |  |  |

Nucleotide position according to *Ry_sto_* (MN393235.1). UTR = Untranslated region, LRR = Leucine-rich repeat motif, TIR = N-terminal domain homologous to the Drosophila Toll domain and human interleukin-1 receptor. Primer pair T does not contain ATG start codon. All primers except pair V were designed in this study using Primer-BLAST [37]. Primer pair V (630-35S-F/630-35S-R) according to Grech Baran et al. (2020) [19]

**Table S9.** PCR reaction mixture and conditions

| PCR reaction mixture and conditions | | | | | | |
| --- | --- | --- | --- | --- | --- | --- |
| **PCR reaction mix for DreamTaq DNA Polymerase (primer pairs A, C, H, I, J, K and RystoF2R3)** | | | | | | |
|  | | | | | | |
| **Component** | | | | **Volume** (Final volume 20 μL ) | | |
| H_2_O | | | | 15 μL | | |
| 10x DreamTaq Buffer | | | | 2 μL | | |
| dNTP mix (2 mM of each) | | | | 1 μL | | |
| Forward primer (0.2 µM) | | | | 0.4 μL | | |
| Reverse primer (0.2 µM) | | | | 0.4 μL | | |
| DreamTaq DNA Polymerase (5 U/µL) | | | | 0.2 μL | | |
|  | | | |  | | |
| **PCR program for primer pairs A, C, H, I, J, K** | | | |  | | |
|  | | | |  | | |
| **Step** | | **Temperature** | | **Time** | | **Cycles** |
| 1 | | 94ºС | | 3 min | |  |
| 2 | | 94ºС | | 30s | | 29 |
| 3 | | 60ºС | | 30s | |  |
| 4 | | 72ºС | | 45s | |  |
| 5 | | 72ºС | | 7 min | |  |
|  | | | | | | |
| **PCR program for primer pair RystoF2R3** | | | | | | |
|  | | | | | | |
| **Step** | | **Temperature** | | **Time** | | **Cycles** |
| 1 | | 93ºС | | 2 min | |  |
| 2 | | 93ºС | | 45s | | 34 |
| 3 | | 55ºС | | 45s | |  |
| 4 | | 72ºС | | 90s | |  |
| 5 | | 72ºС | | 10 min | |  |
|  | | | | | | |
| **PCR reaction mix for Phusion™ High-Fidelity DNA Polymerase (primer pairs T, U, V)** | | | | | | |
|  | | | | | | |
| **Component** | | | | **Volume** (Final volume 20 μL ) | | |
| H2O | | | | 10.8 μL | | |
| 5x Phusion™ HF Buffer | | | | 4 μL | | |
| dNTP mix (2 mM of each) | | | | 2 μL | | |
| Forward primer (0.5 µM) | | | | 1 μL | | |
| Reverse primer (0.5 µM) | | | | 1 μL | | |
| Phusion™ High–Fidelity DNA Polymerase (2 U/µL) | | | | 0.2 μL | | |
|  | | | | | | |
| **PCR program for primer pairs T, U, V** | | | | | | |
|  | | | | | | |
| **Cycle type** | **Temperature** | | **Time** | | **Cycles** | |
| 1 | 98ºС | | 3 min | |  | |
| 2 | 98ºС | | 30s | | 34 | |
| 3 | 61-64ºС | | 30s | |  |  |
| 4 | 72ºС | | 3 - 5 min | |  |  |
| 5 | 72ºС | | 7 - 10 min | |  | |

Phusion™ High-Fidelity DNA Polymerase and DreamTaq DNA Polymerase producer (both Thermo Fisher Scientific Inc., Waltham, MA, USA)

**Table S10.** *Ry_sto_*-specific primer pairs V, U and T with PacBio barcodes

| **Primer name** | **Primer sequence** |
| --- | --- |
|  | /5’Block/5 bp Pad (in gray shadow) + **16 bp Barcode (in bold)** + *Ry_sto_*-specific primer |
| **Barcoded V forward primer** | |
| B1RystoV_For_bc1005 | /5Phos/GCATC**CACTCGACTCTCGCGT**GGTGCTAAGAAGACTTCATATCAG |
| B2RystoV_For_bc1007 | /5Phos/GCATC**TCTGTATCTCTATGTG**GGTGCTAAGAAGACTTCATATCAG |
| B3RystoV_For_bc1008 | /5Phos/GCATC**ACAGTCGAGCGCTGCG**GGTGCTAAGAAGACTTCATATCAG |
| **Barcoded V reverse primer** | |
| B4RystoV_Rev_bc1033 | /5Phos/GCATC**AGAGACTGCGACGAGA**GGATACTCAACGTATCTACCTTATTATAA |
| B5RystoV_Rev_bc1035 | /5Phos/GCATC**CAGAGAGTGCGCGCGC**GGATACTCAACGTATCTACCTTATTATAA |
| B6RystoV_Rev_bc1044 | /5Phos/GCATC**CGCGCGTCGTCTCAGC**GGATACTCAACGTATCTACCTTATTATAA |
| B7RystoV_Rev_bc1045 | /5Phos/GCATC**AGAGAGTACGATATGT**GGATACTCAACGTATCTACCTTATTATAA |
| B8RystoV_Rev_bc1054 | /5Phos/GCATC**TCTGTAGTGCGTGCGC**GGATACTCAACGTATCTACCTTATTATAA |
| B9RystoV_Rev_bc1056 | /5Phos/GCATC**ATGTGCGTGTGTGTCT**GGATACTCAACGTATCTACCTTATTATAA |
| B10RystoV_Rev_bc1057 | /5Phos/GCATC**CTCTCAGACGCTCGTC**GGATACTCAACGTATCTACCTTATTATAA |
| B11RystoV_Rev_bc1059 | /5Phos/GCATC**TATCTCAGTGCGTGTG**GGATACTCAACGTATCTACCTTATTATAA |
| B12RystoV_Rev_bc1060 | /5Phos/GCATC**TGTGTCTATACTCATC**GGATACTCAACGTATCTACCTTATTATAA |
| B13RystoV_Rev_bc1062 | /5Phos/GCATC**TATAGACTATCTGAGA**GGATACTCAACGTATCTACCTTATTATAA |
| B14RystoV_Rev_bc1065 | /5Phos/GCATC**GTATGTGAGAGAGCGC**GGATACTCAACGTATCTACCTTATTATAA |
| B15RystoV_Rev_bc1075 | /5Phos/GCATC**CACGCGACGCTCTCTA**GGATACTCAACGTATCTACCTTATTATAA |
| **Barcoded U forward primer** | |
| B31RystoU_For_bc1022 | /5Phos/GCATC**CACTCACGTGTGATAT**TGTTGCAGACAGAGTGTGGA |
| B32RystoU_For_bc1024 | /5Phos/GCATC**CATGTAGAGCAGAGAG**TGTTGCAGACAGAGTGTGGA |
| **Barcoded U reverse primer** | |
| B33RystoU_Rev_bc1086 | /5Phos/GCATC**GCGCTCTATGCGCGAC**CGTGTTTGGTTGAGTACGGA |
| B34RystoU_Rev_bc1087 | /5Phos/GCATC**TGTGAGTGCTAGATAG**CGTGTTTGGTTGAGTACGGA |
| B35RystoU_Rev_bc1088 | /5Phos/GCATC**CACGCAGAGTGACACG**CGTGTTTGGTTGAGTACGGA |
| B36RystoU_Rev_bc1091 | /5Phos/GCATC**AGACGTATATATATAC**CGTGTTTGGTTGAGTACGGA |
| B37RystoU_Rev_bc1095 | /5Phos/GCATC**CGCGCTGACATCGTGC**CGTGTTTGGTTGAGTACGGA |
| B38RystoU_Rev_bc1097 | /5Phos/GCATC**TAGAGAGATAGAGACG**CGTGTTTGGTTGAGTACGGA |
| B39RystoU_Rev_bc1103 | /5Phos/GCATC**CGAGCGAGTGTGTATA**CGTGTTTGGTTGAGTACGGA |
| B40RystoU_Rev_bc1107 | /5Phos/GCATC**ATATATGCACGCTCTA**CGTGTTTGGTTGAGTACGGA |
| B41RystoU_Rev_bc1109 | /5Phos/GCATC**ACTCTATCACACACAG**CGTGTTTGGTTGAGTACGGA |
| B42RystoU_Rev_bc1114 | /5Phos/GCATC**TGTACACGCTACTCTC**CGTGTTTGGTTGAGTACGGA |
| B43RystoU_Rev_bc1117 | /5Phos/GCATC**ATATGTATGTATGCAC**CGTGTTTGGTTGAGTACGGA |
| B44RystoU_Rev_bc1118 | /5Phos/GCATC**AGTATCATGTGTATCT**CGTGTTTGGTTGAGTACGGA |
| **Barcoded T forward primer** | |
| B16RystoT_For_bc1012 | /5Phos/GCATC**ACACTAGATCGCGTGT**TCTCATGCTTCTTCTTCCAAAGT |
| B17RystoT_For_bc1015 | /5Phos/GCATC**CGCATGACACGTGTGT**TCTCATGCTTCTTCTTCCAAAGT |
| B18RystoT_For_bc1020 | /5Phos/GCATC**CACGACACGACGATGT**TCTCATGCTTCTTCTTCCAAAGT |
| **Barcoded T reverse primer** | |
| B19RystoT_Rev_bc1077 | /5Phos/GCATC**CGCGACTCGAGAGATA**ACCTTTGCATCACTCATTGCTT |
| B20RystoT_Rev_bc1078 | /5Phos/GCATC**GACGTGTATGTGTGAG**ACCTTTGCATCACTCATTGCTT |
| B21RystoT_Rev_bc1079 | /5Phos/GCATC**ACACAGAGTGTACTAT**ACCTTTGCATCACTCATTGCTT |
| B22RystoT_Rev_bc1082 | /5Phos/GCATC**GTGCTCTGTGTGTCAC**ACCTTTGCATCACTCATTGCTT |
| B23RystoT_Rev_bc1084 | /5Phos/GCATC**GCACAGACGAGAGACG**ACCTTTGCATCACTCATTGCTT |
| B24RystoT_Rev_bc1085 | /5Phos/GCATC**GCTCGCTCTACTGTGT**ACCTTTGCATCACTCATTGCTT |
| B25RystoT_Rev_bc1093 | /5Phos/GCATC**TCTACTCTCGCATCTA**ACCTTTGCATCACTCATTGCTT |
| B26RystoT_Rev_bc1094 | /5Phos/GCATC**AGAGAGATGTCGCTAT**ACCTTTGCATCACTCATTGCTT |
| B27RystoT_Rev_bc1096 | /5Phos/GCATC**CTGTGTAGAGAGCACA**ACCTTTGCATCACTCATTGCTT |
| B28RystoT_Rev_bc1102 | /5Phos/GCATC**CTATCATATCGAGAGA**ACCTTTGCATCACTCATTGCTT |
| B29RystoT_Rev_bc1106 | /5Phos/GCATC**AGAGCATATAGTCGTG**ACCTTTGCATCACTCATTGCTT |
| B30RystoT_Rev_bc1108 | /5Phos/GCATC**TGTCAGTGCGCGTCGT**ACCTTTGCATCACTCATTGCTT |

The barcode for reverse primer is in reverse complement direction
