# Supplementary material for: Diversity of the Rysto gene conferring resistance to potato virus Y in wild relatives of potato
Source: BMC Plant Biol. 2024 May 8;24:375. doi: 10.1186/s12870-024-05089-2 (PMC11077776; doi:10.1186/s12870-024-05089-2)
Supplement: Supplementary file 5 — Additional file 5: Figure S1. Screening for the presence of Rysto homologues in wild relatives of potato. In total, 298 potato genotypes representing 29 accessions of 26 tuber-bearing Solanum species were examined. Six resistant control genotypes, potato cultivars Alicja, Bzura, Hinga, Nimfy, White Lady and the breeding line PW363, were included. (A) Number of potato genotypes in which fragments of the Rysto gene were detected using primer pairs A, C and J. (B) Number of potato genotypes in which the full coding sequence of the Rysto gene were detected using primer pairs T, U and V. (C) Number of potato genotypes in which the Rysto homologues were obtained using the barcoded primer pairs T, U and V. (D) A schematic view of the Rysto gene, indicating the different target regions amplified by primer pairs A, C, J, T, U and V. Exons are shown as rectangles with colour-coded domains: UTR = untranslated region, TIR = N-terminal domain homologous to the Drosophila Toll domain and human interleukin-1 receptor, NB-ARC = nucleotide-binding domain, LRR = leucine-rich repeat motif, C-JID = C-terminal jelly roll/Ig-like domain. [file 12870_2024_5089_MOESM5_ESM.pdf]

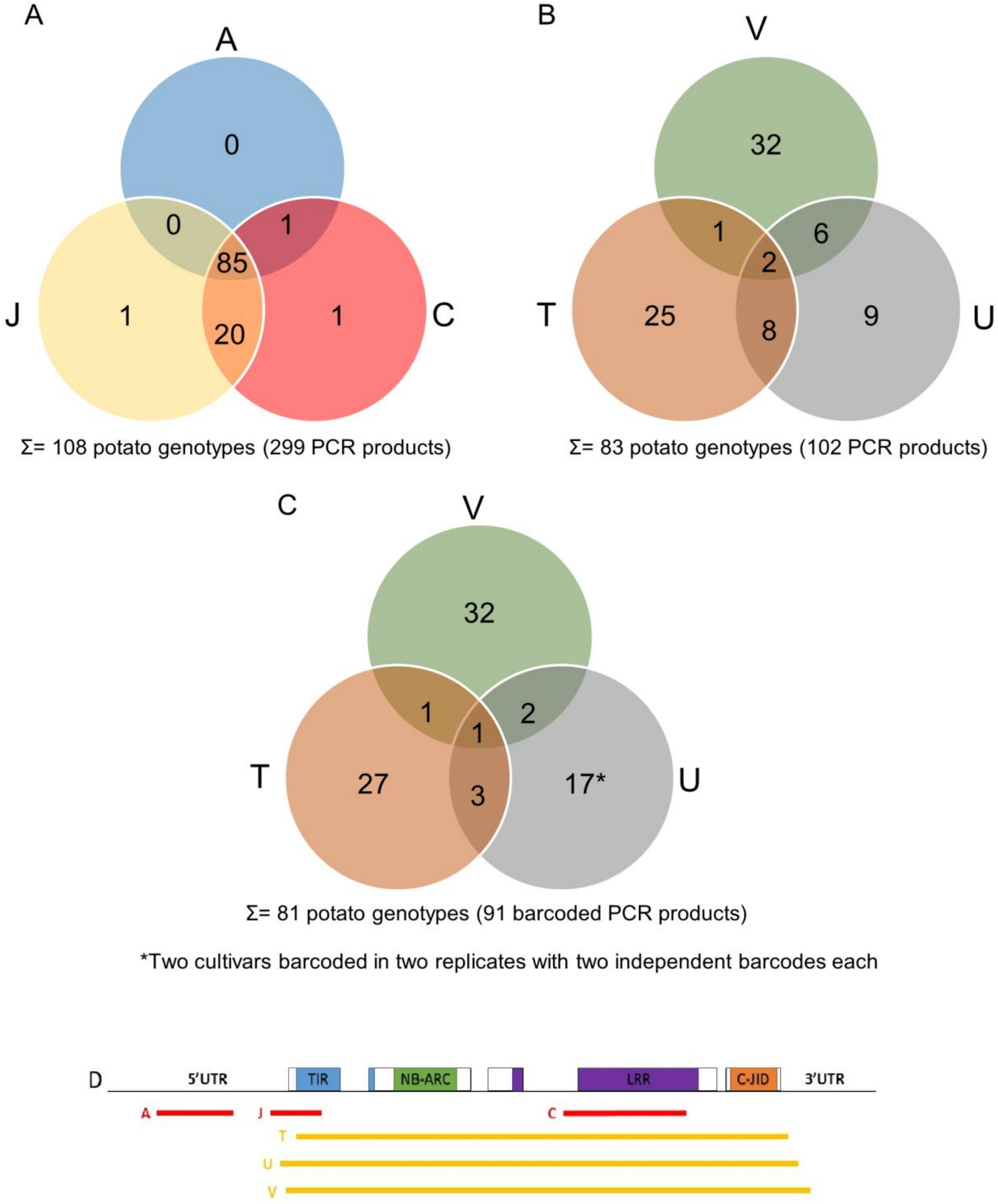

A
A
0
0
1
85
J
1
20
1
C
Σ= 108 potato genotypes (299 PCR products)
B
V
32
1
6
2
T
25
8
9
U
Σ= 83 potato genotypes (102 PCR products)
C
V
32
1
2
1
T
27
3
17*
U
Σ= 81 potato genotypes (91 barcoded PCR products)
*Two cultivars barcoded in two replicates with two independent barcodes each
D
5'UTR
TIR
NB-ARC
LRR
C-JID
3'UTR
A
J
C
T
U
V
