# Supplementary figures and images for: Diversity of the Rysto gene conferring resistance to potato virus Y in wild relatives of potato
Source: BMC Plant Biol. 2024 May 8;24:375. doi: 10.1186/s12870-024-05089-2 (PMC11077776; doi:10.1186/s12870-024-05089-2)

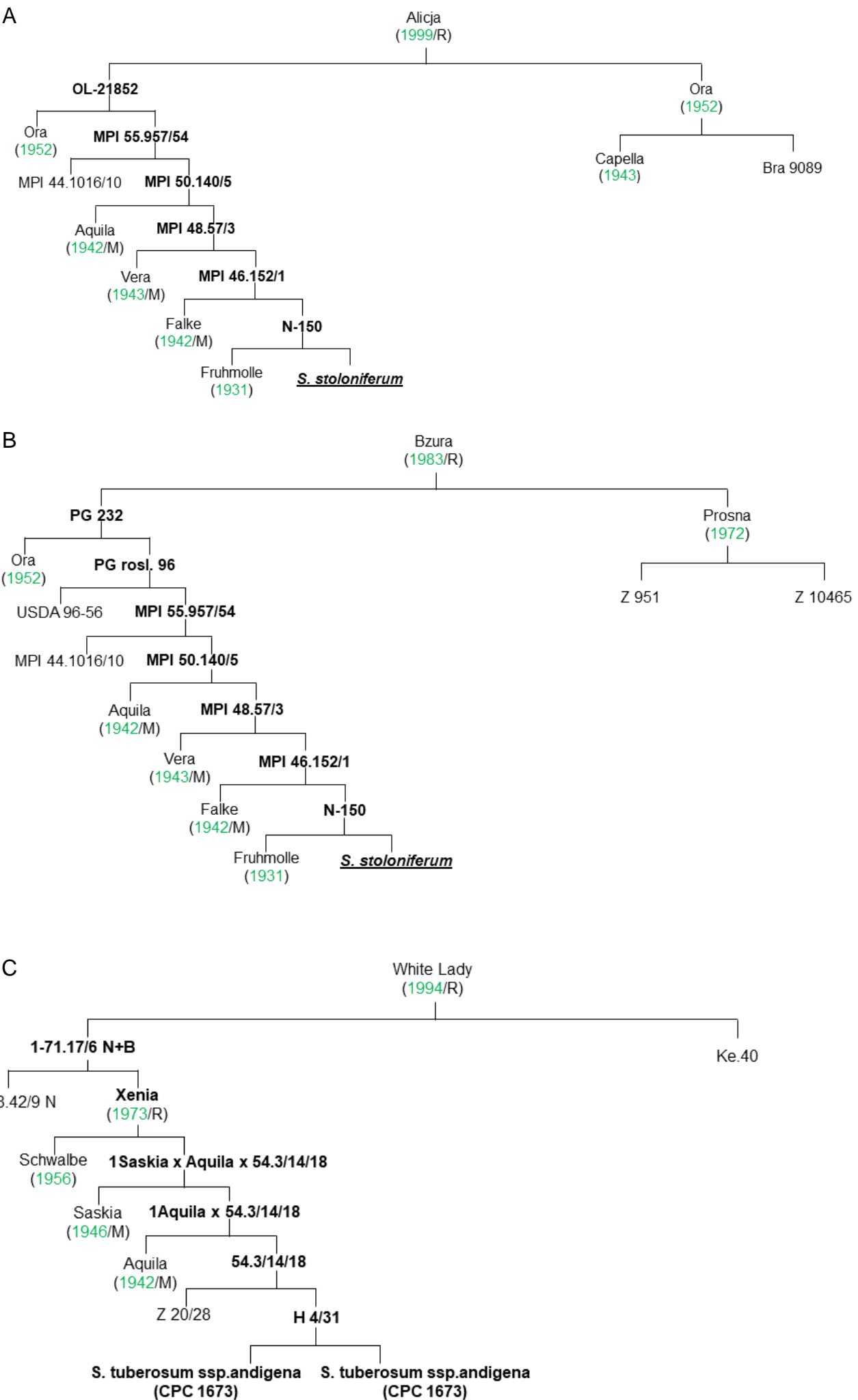

D

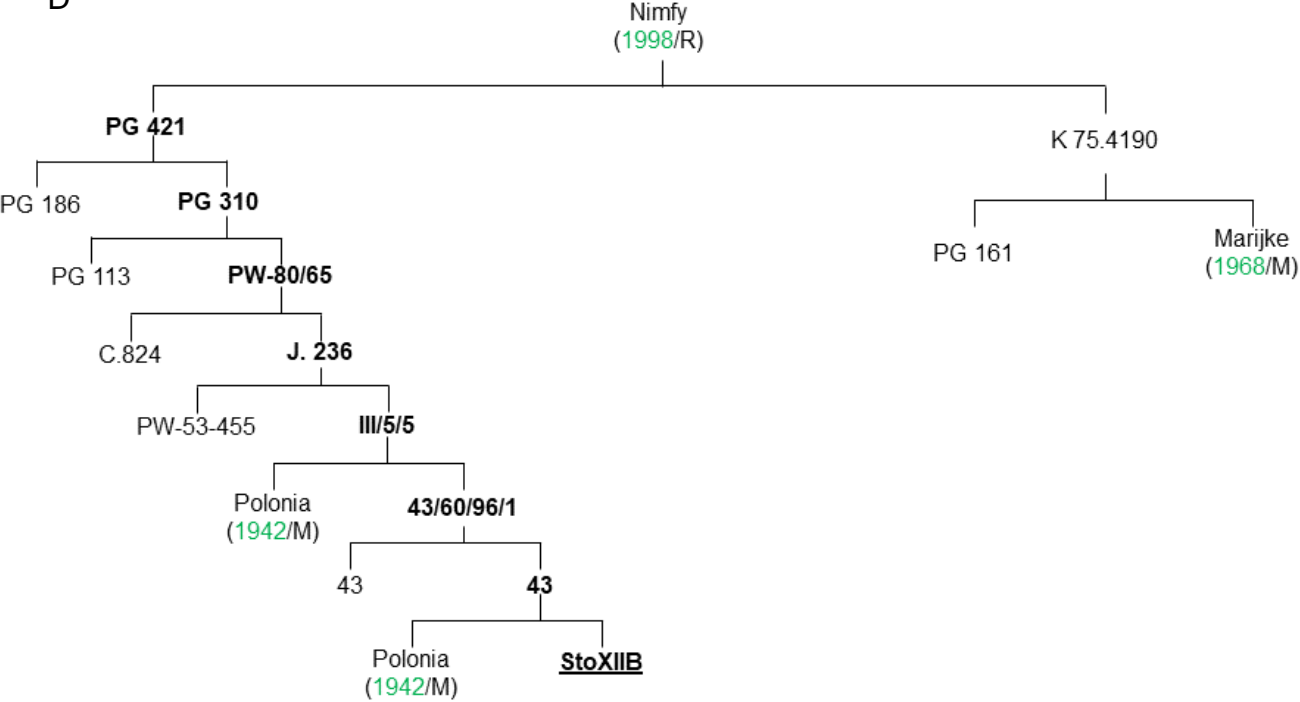

E

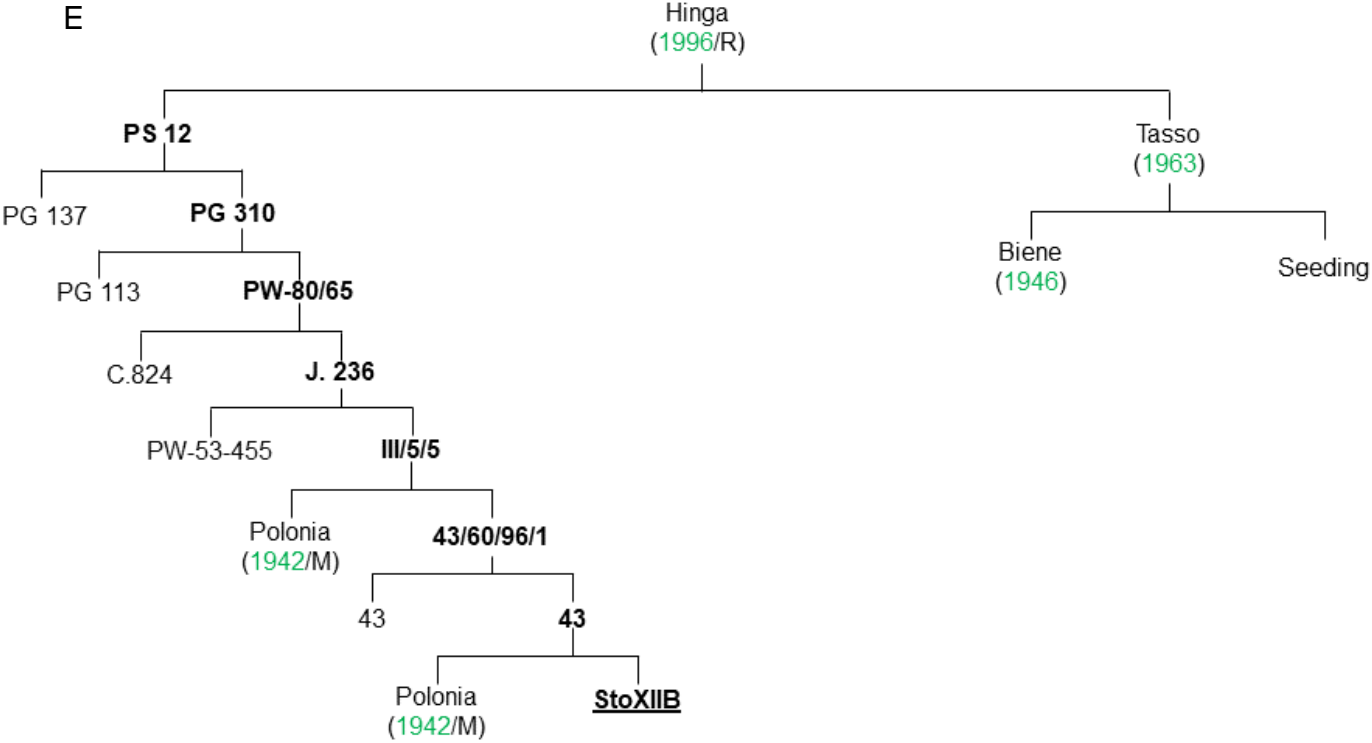

F

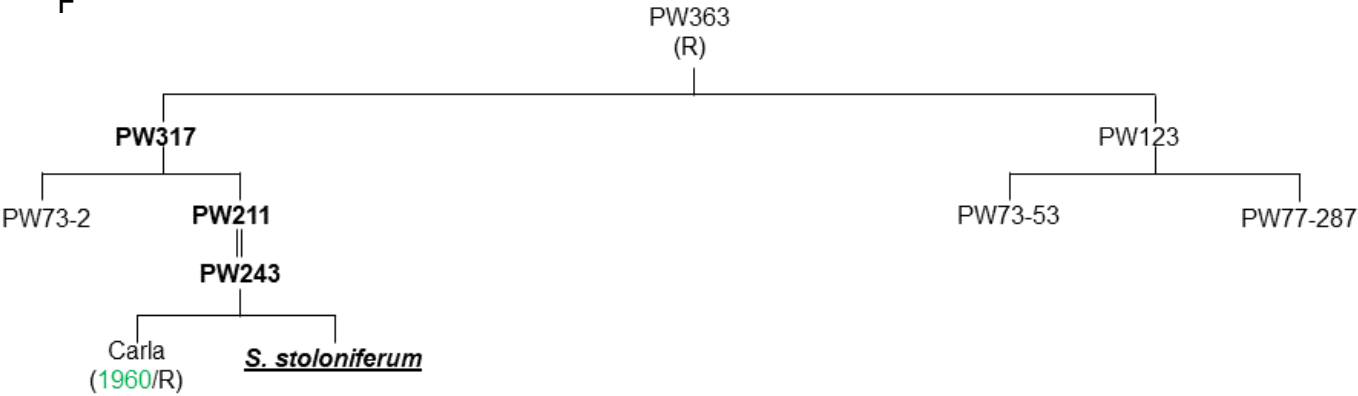

Supplement: Supplementary file 6 — Additional file 6: Figure S2. Pedigrees of the resistant potato controls. (A) Alicja. (B) Bzura. (C) White Lady. (D) Nimfy. (E) Hinga. (F) breeding line PW363. The year of registration of the cultivars and level of resistance to potato virus Y (PVY) are given in brackets (R = resistant, M = medium resistant). The likely source of PVY resistance is in bold. StoXIIB is the name of a S. stoloniferum clone from Plant Breeding and Acclimatization Institute – National Research Institute, Poland. [file 12870_2024_5089_MOESM6_ESM.pdf]
